# Supplementary figures and images for: Nr4a1-Dependent Ly6Clow Monocytes Monitor Endothelial Cells and Orchestrate Their Disposal
Source: Cell. 2013 Apr 11;153(2):362–75. doi: 10.1016/j.cell.2013.03.010 (PMC3898614; doi:10.1016/j.cell.2013.03.010)

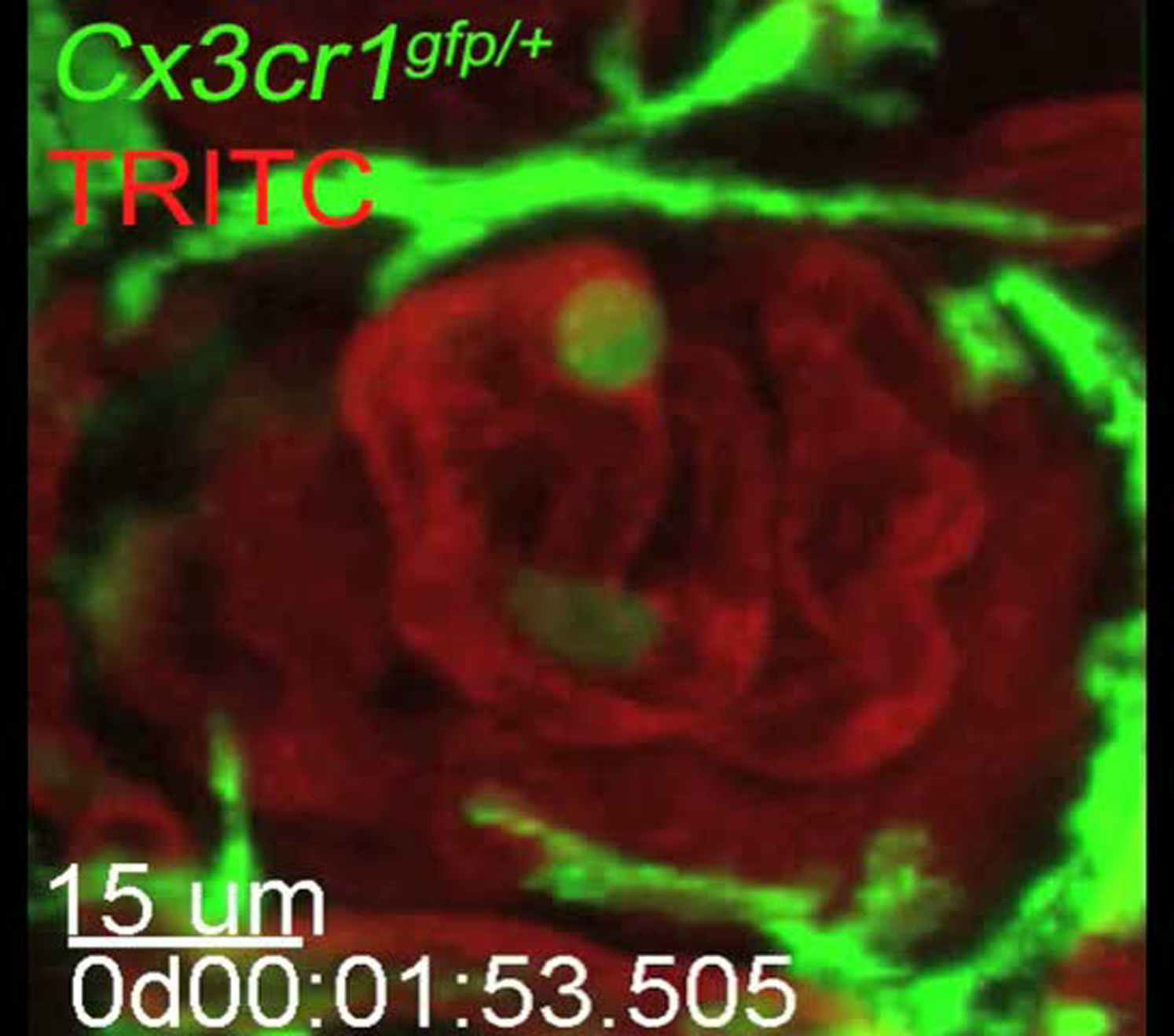

Supplement: Movie S1. Monocyte Intravascular Crawling in the Kidney, Related to Figure 1 — The first sequence shows time-lapse intravital microscopy of the peritubular capillaries in the superficial renal cortex of a Cx3cr1gfp/+ mouse. The movie is shown at 120× real time. Green, GFP; red, i.v. injected; TRITC, 70 kd dextran. White arrows point out monocyte crawling intravascularly. The second sequence shows time-lapse intravital microscopy of a glomerulus depicting glomerular capillaries in the superficial renal cortex of a Cx3cr1gfp/+ mouse. The movie is shown at 120× real time. Green, GFP; red, i.v. injected; TRITC, 70 kd dextran. [file mmc1.jpg]

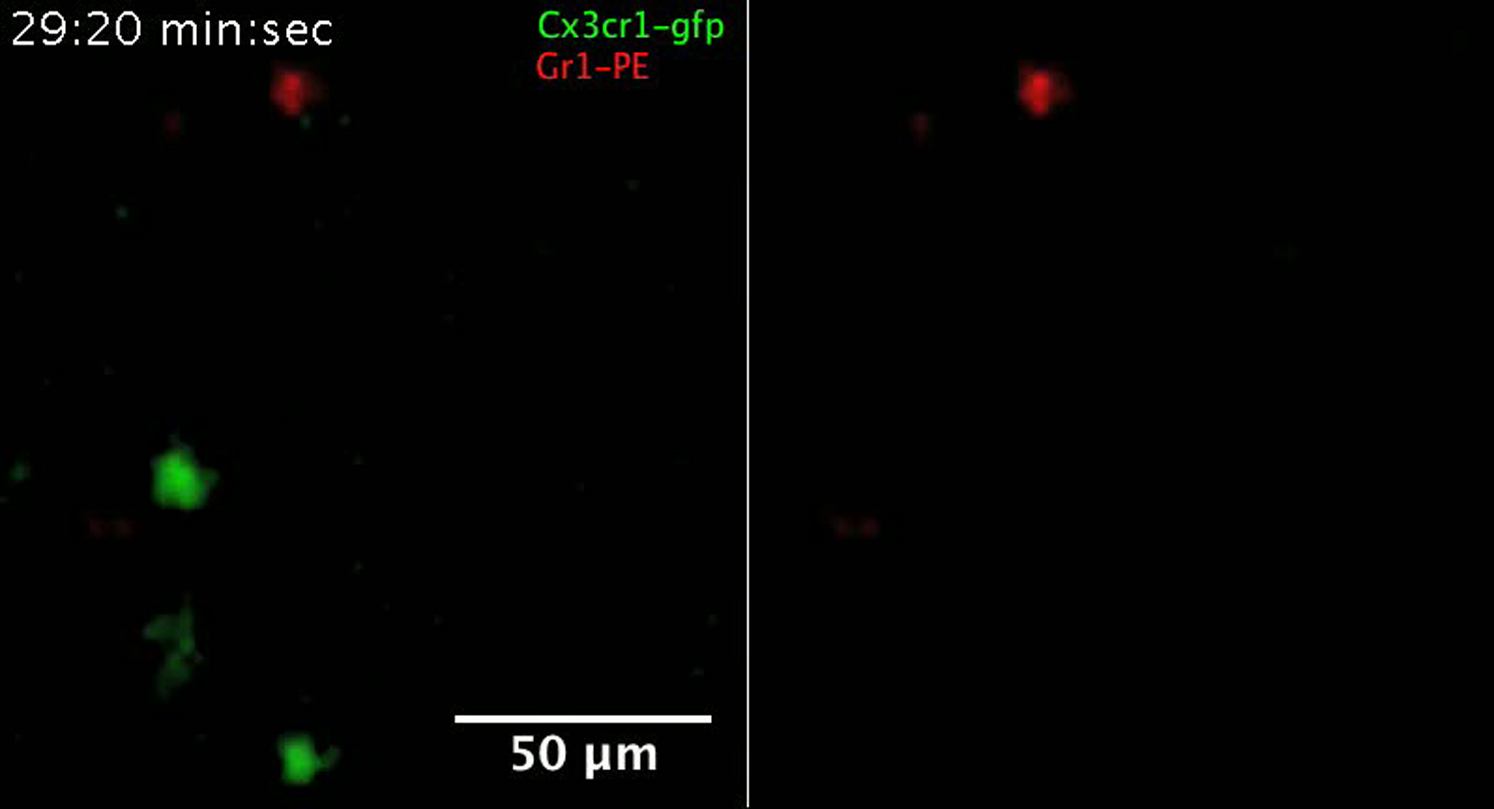

Supplement: Movie S2. Attachment, Related to Figure 1 — Microscopy of a mesenteric venule in a Cx3cr1gfp/+ mouse. Intravital antibody labeling. The movie is shown at 120× real time. Gr1-GFP+ Monocyte attaches and crawls on the lumenal side of the vascular endothelium. Green, GFP; red, i.v. injected PE-anti-Gr1 (RB6-8c5). The white circle shows where monocyte attaches. [file mmc2.jpg]

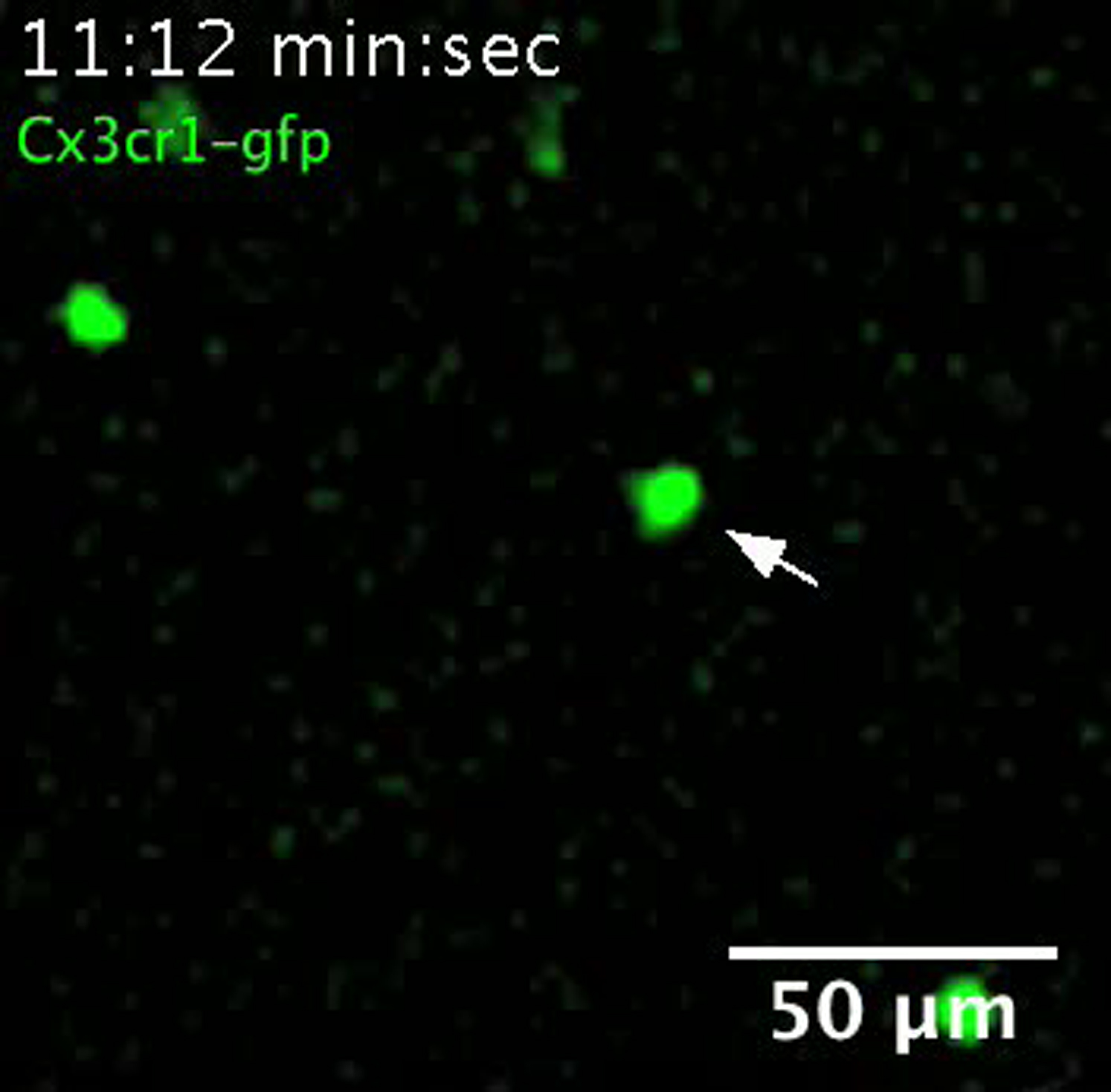

Supplement: Movie S3. Detachment, Related to Figure 1 — Time-lapse intravital microscopy of a mesenteric venule in a Cx3cr1gfp/+ mouse. The movie is shown at 120× real time. The GFP+ monocyte crawls and then detaches from the lumenal side of the vascular endothelium. Green, GFP. The white arrow points out monocyte that detaches. [file mmc3.jpg]

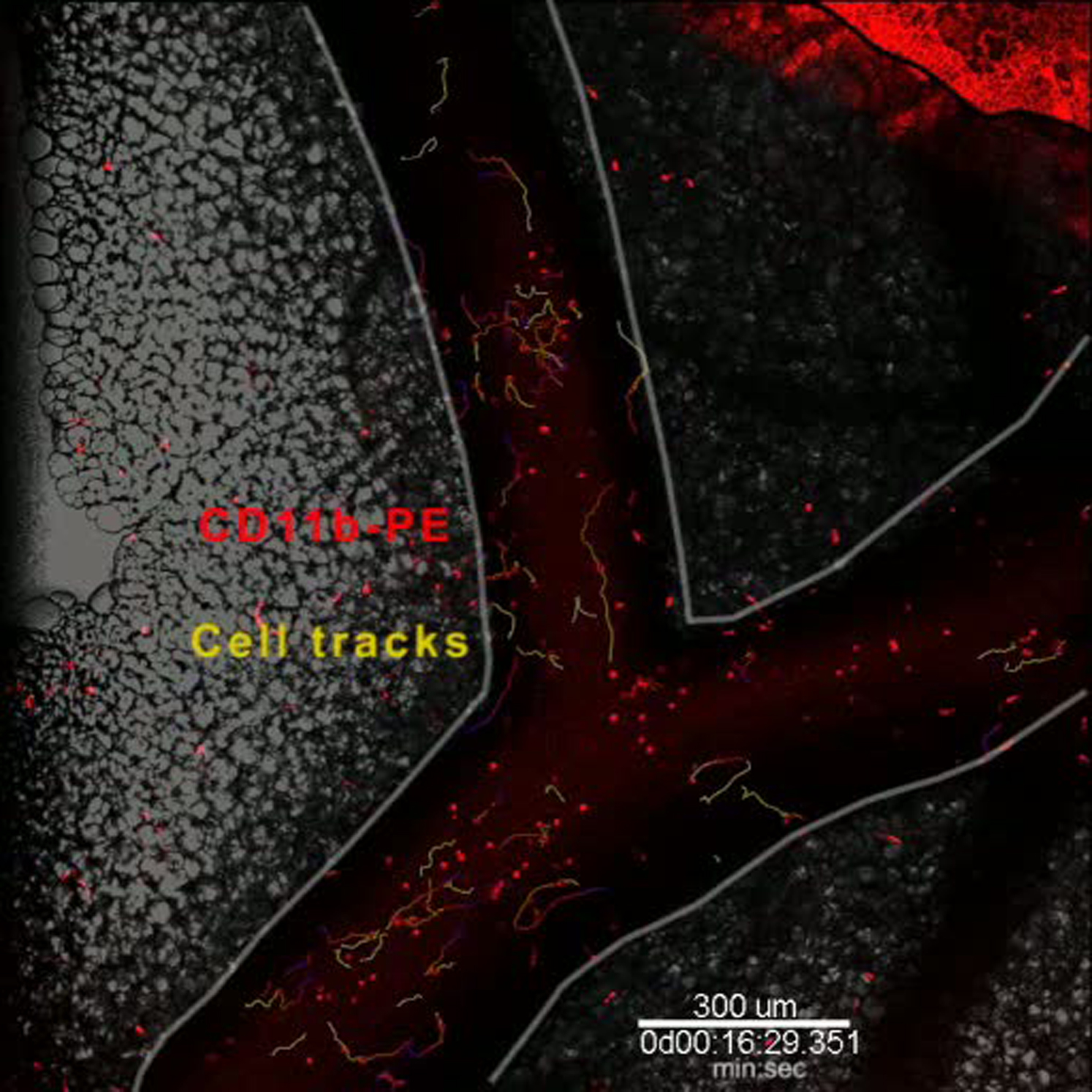

Supplement: Movie S4. Intravital Labeling and Cell Tracking, Related to Figure 1 — Time-lapse intravital microscopy of a mesenteric venule in a Cx3cr1gfp/+mouse. The movie is shown at 120× real time. Shown are Cx3cr1-gfp (green) alone, then Cx3cr1-gfp (green) and i.v. injected APC-anti-Gr1 (RB6-8c5; cyan), then i.v. injected PE-anti-CD11b (red) alone. Cell tracks are subsequently shown overlaid on the movie. Finally, the track analysis process is shown schematically. [file mmc4.jpg]

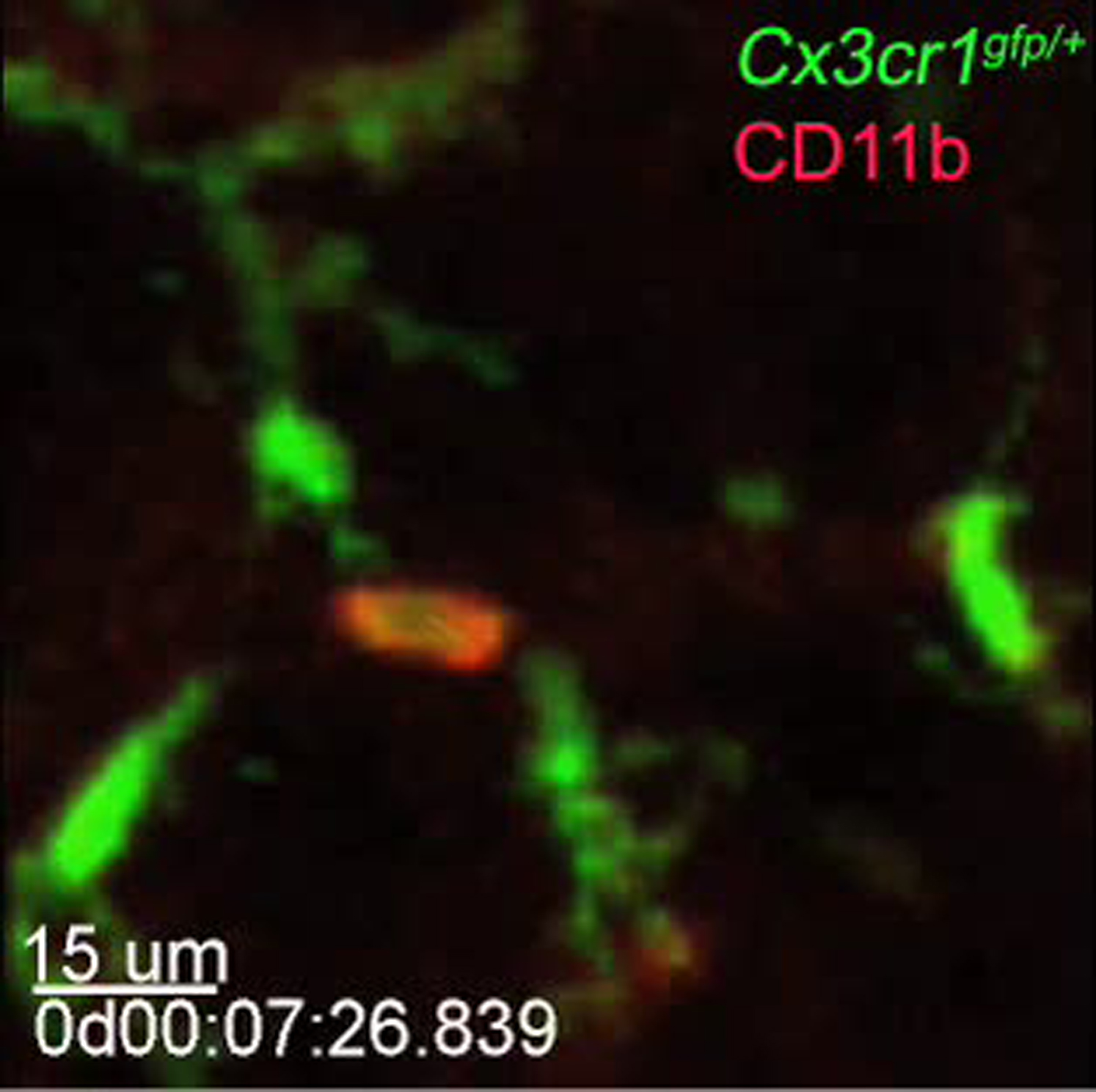

Supplement: Movie S5. Mac 1 Expression, Related to Figure 1 — Time-lapse intravital microscopy of the peritubular capillaries in the superficial renal cortex of a Cx3cr1gfp/+mouse. The movie is shown at 120× real time. The monocyte is labeled with i.v. injected anti-CD11b. Green, GFP; red, i.v. injected PE-anti-CD11b. The white arrow points out monocyte crawling intravascularly. [file mmc5.jpg]

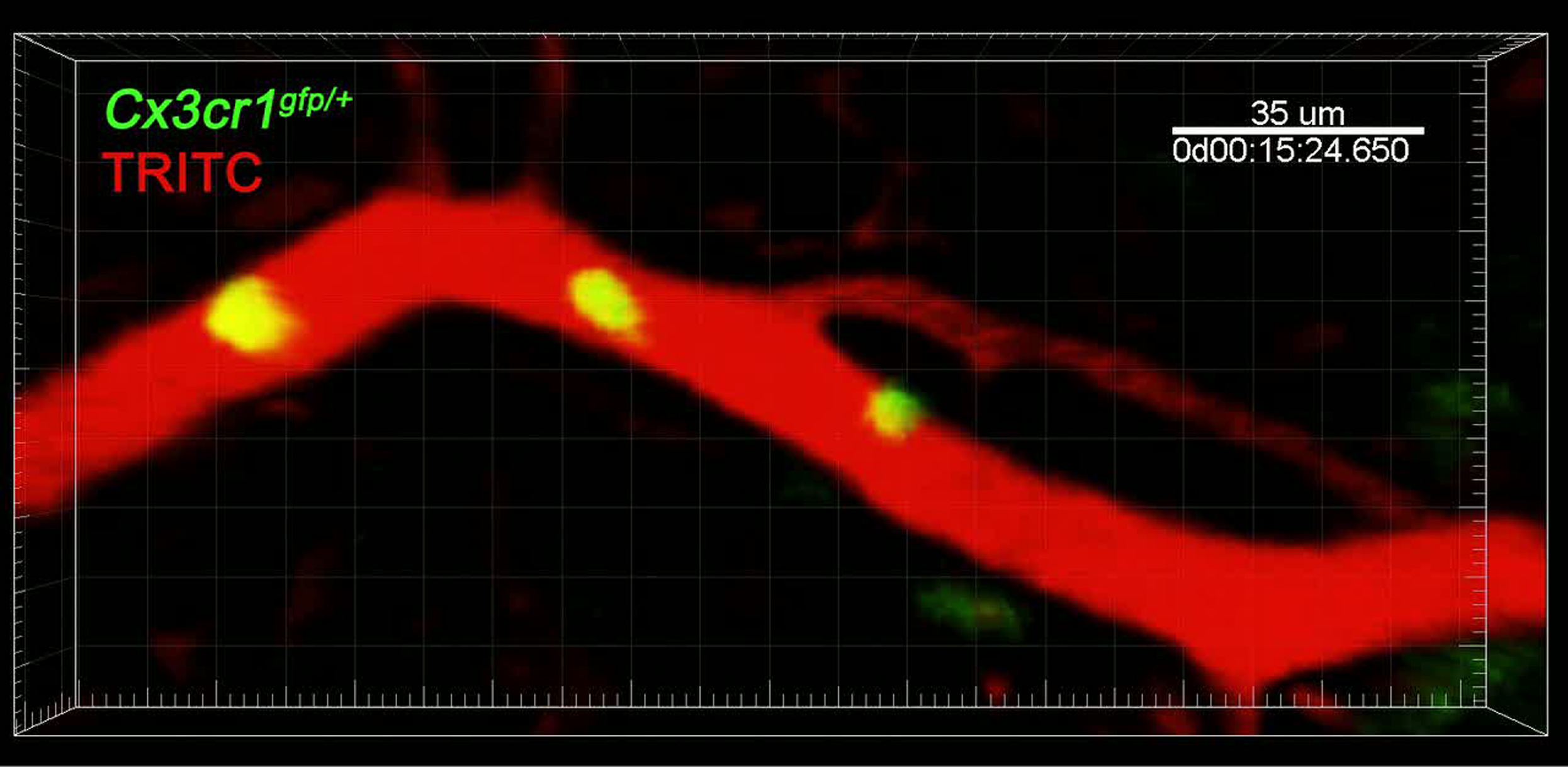

Supplement: Movie S6. Filopodia In Vivo, Related to Figure 1 — Time-lapse intravital microscopy of dermal blood vessels in the ear of a Cx3cr1gfp/+mouse. The movie is shown at 120× real time. The data originally presented in Auffray et al. 2007 were deconvolved with Autoquant blind deconvolution (using settings for confocal microscopy) to increase spatial resolution and then projected to reveal and emphasize “dendritic” appearance of crawling monocytes. Green, GFP; red, i.v. injected; TRITC, 70 kD dextran. [file mmc6.jpg]

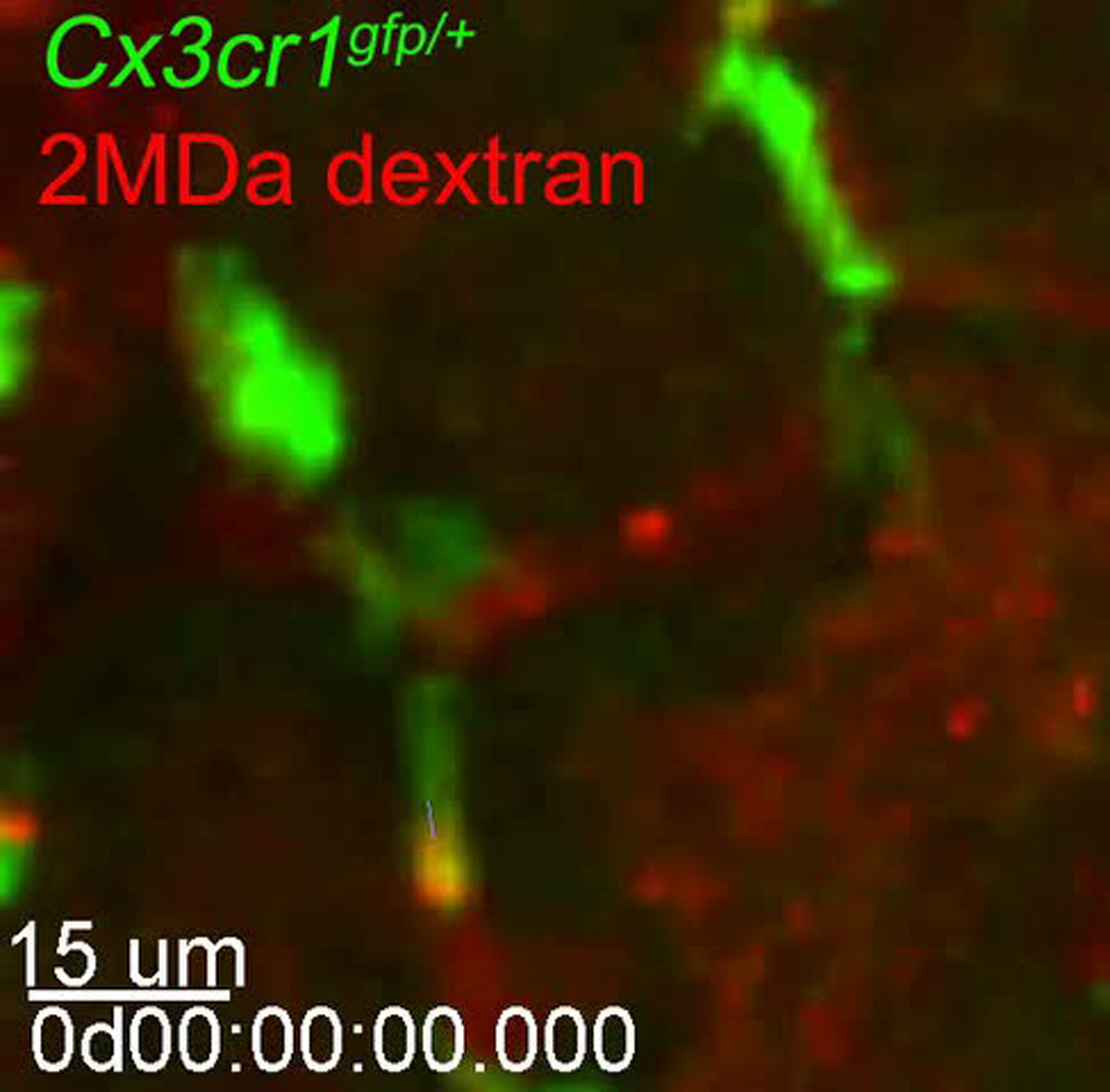

Supplement: Movie S7. Monocyte Scavenging, Related to Figure 1 — The first two sequences show time-lapse intravital microscopy of peritubular capillaries in the superficial renal cortex of a Cx3cr1gfp/+mouse where 2 μm TRITC-labeled latex beads have been i.v. injected and associate with the endothelium. The white circle points out a bead which is scavenged by a monocyte, which then continues to patrol. The movie is shown at 120× real time. Green, GFP; red, TRITC-labeled 2 μm latex bead. The third sequence shows – time-lapse intravital microscopy of a peritubular capillary in the superficial renal cortex of a Cx3cr1gfp/+mouse. The movie is shown at 120× real time. A monocyte scavenges TRITC dextran from the endothelium. Green, GFP; red, i.v. injected; TRITC, 2 MDa dextran. [file mmc7.jpg]

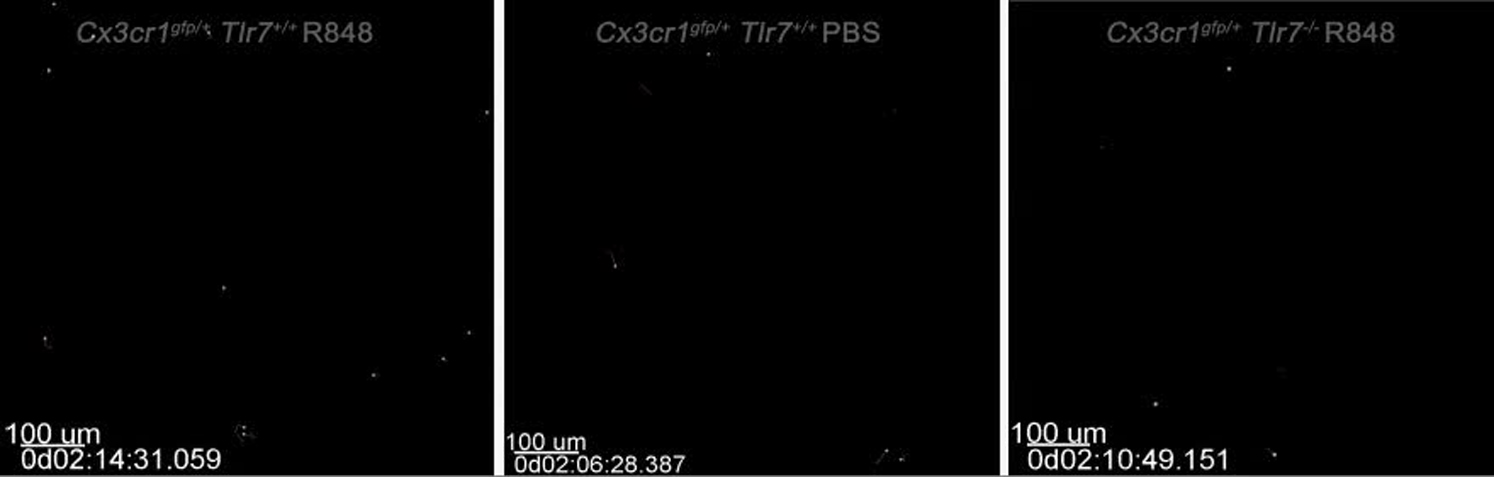

Supplement: Movie S8. Monocyte Retention by Capillaries via TLR7, Related to Figure 3 — Analysis of time-lapse intravital microscopy of peritubular capillaries in the superficial renal cortex of Cx3cr1gfp/+, Tlr7+/+, or Tlr7−/− mice 0–5 hr after direct treatment of kidney capsule with R848 or PBS. Each dot represents a single tracked monocyte. The movie is shown at 120× real time. [file mmc8.jpg]

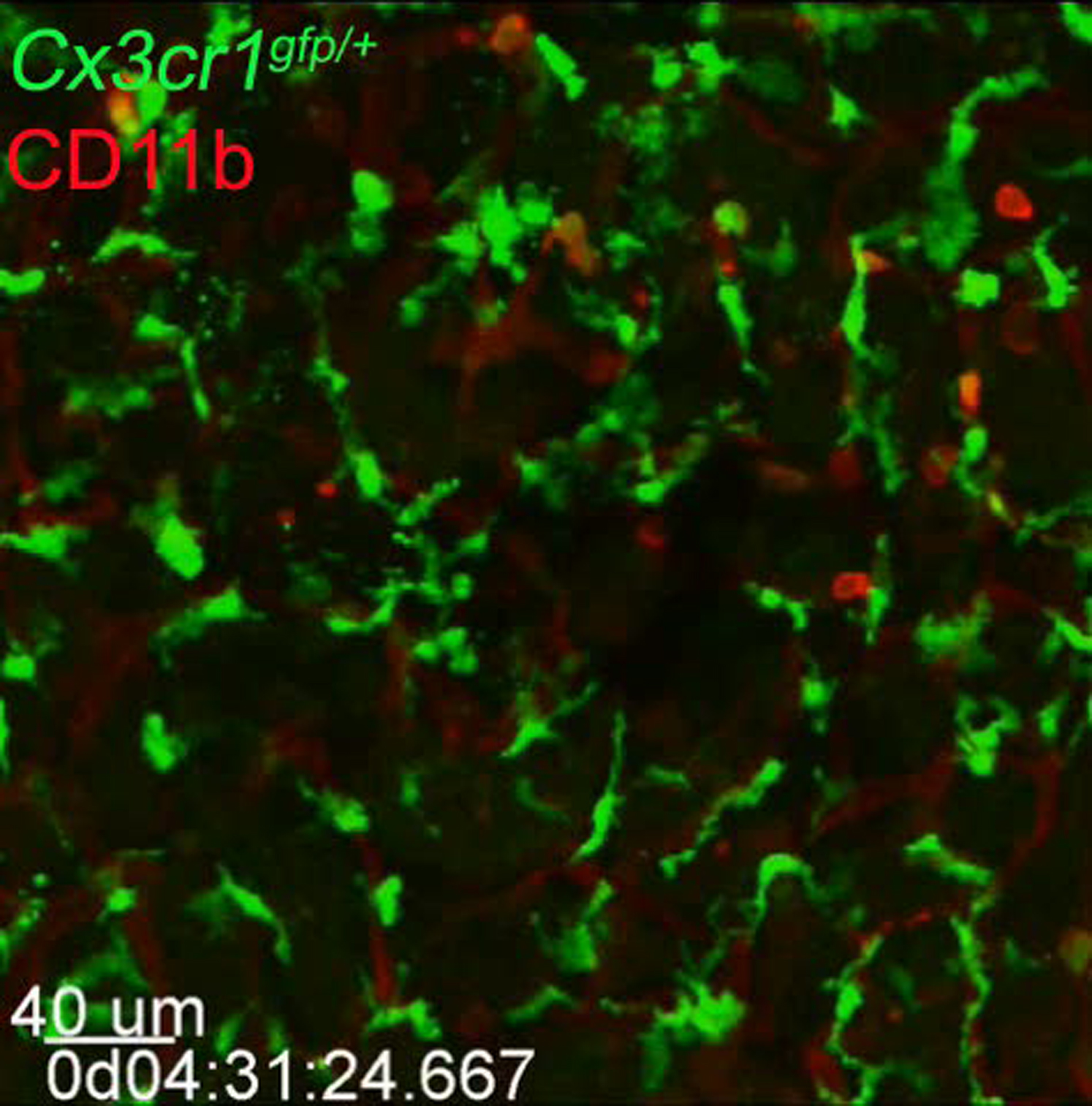

Supplement: Movie S9. Intravascular Retention, Related to Figure 3 — Time-lapse intravital microscopy of peritubular capillaries in the superficial renal cortex of a Cx3cr1gfp/+mouse from 0–5 hr after direct treatment of the kidney with R848. At 4.5 hr, PE-labeled anti-CD11b is i.v. injected and labels the monocytes instantaneously showing that they remain intravascular. The movie is shown at 120× real time. Green, GFP; red, PE-anti-CD11b. [file mmc9.jpg]

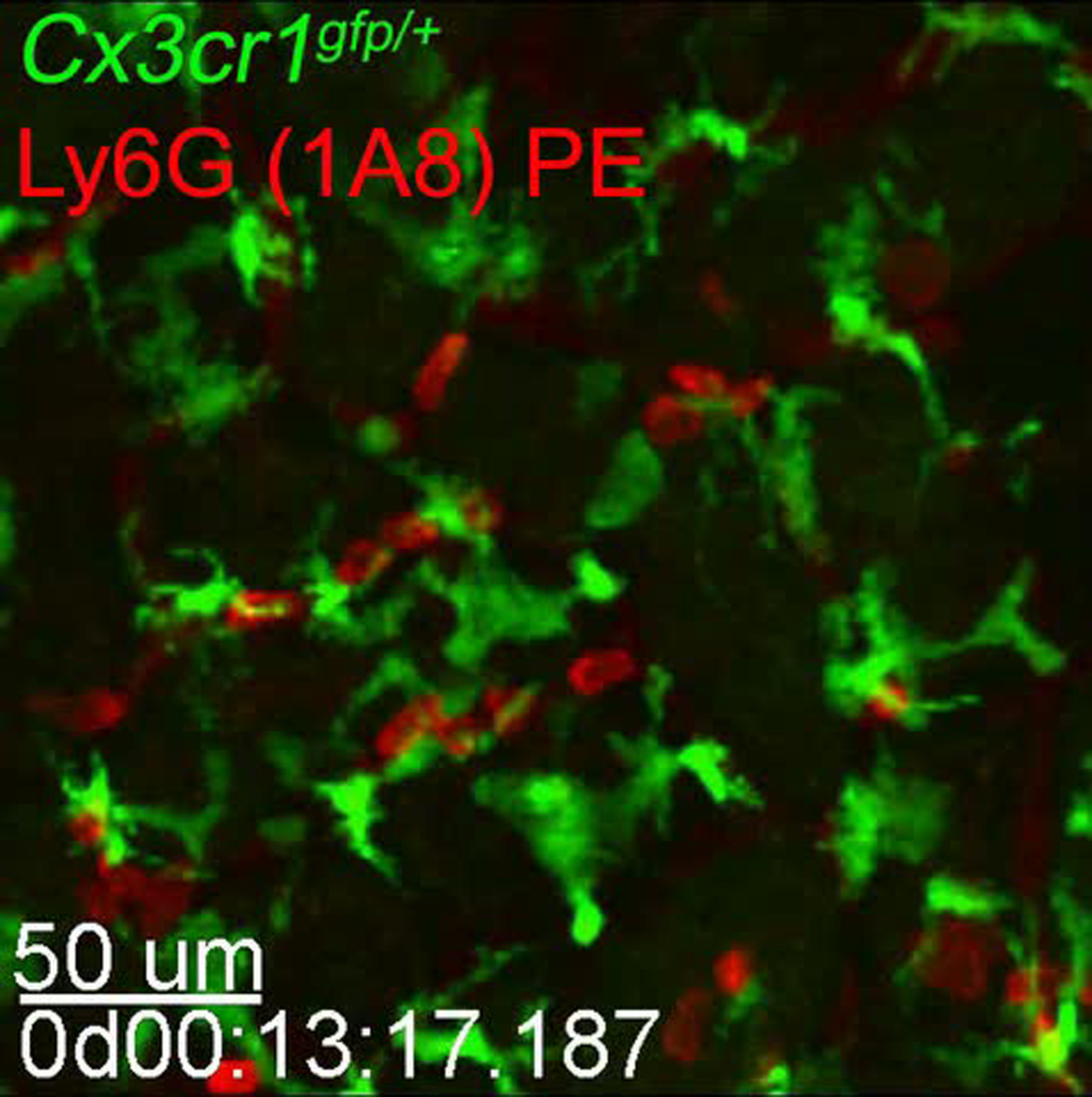

Supplement: Movie S10. Neutrophil Recruitment, Related to Figure 4 — Time-lapse intravital microscopy of peritubular capillaries in the superficial renal cortex of a Cx3cr1gfp/+mouse 4.5 hr after kidney painting with R848. I.v. injected anti-Ly6G label neutrophils. The movie is shown at 120× real time. Green, GFP; red, PE-anti-Ly6G. [file mmc10.jpg]
