# Supplementary material for: Nr4a1-Dependent Ly6Clow Monocytes Monitor Endothelial Cells and Orchestrate Their Disposal
Source: Cell. 2013 Apr 11;153(2):362–75. doi: 10.1016/j.cell.2013.03.010 (PMC3898614; doi:10.1016/j.cell.2013.03.010)
Supplement: Document S11. Article plus Supplemental Information [file mmc11.pdf]

Cell

Nr4a1-Dependent Ly6C^{low} Monocytes Monitor Endothelial Cells and Orchestrate Their Disposal

Leo M. Carlin,[1,2,7] Efstathios G. Stamatiades,[1,2,7] Cedric Auffray,[4,8] Richard N. Hanna,[5] Leanne Glover,[3] Gema Vizcay-Barrena,[3] Catherine C. Hedrick,[5] H. Terence Cook,[6] Sandra Diebold,[2] and Frederic Geissmann[1,2,4,*]
[1]Centre for Molecular and Cellular Biology of Inflammation
[2]Peter Gorer Department of Immunobiology
[3]Centre for Ultrastructural Imaging
King's College London, London SE1 1UL, UK
[4]Institut National de la Santé et de la Recherche Médicale (INSERM) U838, Institut Necker, Paris Descartes University, 75015 Paris, France
[5]Division of Inflammation Biology, La Jolla Institute for Allergy and Immunology, La Jolla, CA 92037, USA
[6]Centre for Complement and Inflammation Research, Imperial College London, London W12 0NN, UK
[7]These authors contributed equally to this work
[8]Present address: CNRS UMR8104, INSERM U1016, Institut Cochin, Paris Descartes University, 75014 Paris, France
*Correspondence: frederic.geissmann@kcl.ac.uk
http://dx.doi.org/10.1016/j.cell.2013.03.010

SUMMARY

The functions of *Nr4a1*-dependent Ly6C^{low} monocytes remain enigmatic. We show that they are enriched within capillaries and scavenge microparticles from their lumenal side in a steady state. In the kidney cortex, perturbation of homeostasis by a TLR7-dependent nucleic acid "danger" signal, which may signify viral infection or local cell death, triggers Gα*i*-dependent intravascular retention of Ly6C^{low} monocytes by the endothelium. Then, monocytes recruit neutrophils in a TLR7-dependent manner to mediate focal necrosis of endothelial cells, whereas the monocytes remove cellular debris. Prevention of Ly6C^{low} monocyte development, crawling, or retention in *Nr4a1*$^{-/-}$, *Itgal*$^{-/-}$, and *Tlr7*$^{host-/-BM+/+}$ and *Cx3cr1*$^{-/-}$ mice, respectively, abolished neutrophil recruitment and endothelial killing. Prevention of neutrophil recruitment in *Tlr7*$^{host+/+BM-/-}$ mice or by neutrophil depletion also abolished endothelial cell necrosis. Therefore, Ly6C^{low} monocytes are intravascular housekeepers that orchestrate the necrosis by neutrophils of endothelial cells that signal a local threat sensed via TLR7 followed by the in situ phagocytosis of cellular debris.

INTRODUCTION

Monocytes are a heterogeneous population of blood phagocytic leucocytes that differentiate in the bone marrow. Inflammatory signals, such as chemokines, promote leucocyte diapedesis into damaged and infected tissues in order to recruit neutrophils within a few hours and "inflammatory" lymphocyte antigen 6c (Ly6C)$^{+}$ monocytes 1 day later, herein initiating a cellular immune response (Auffray et al., 2009b; Serbina et al., 2008). Ly6C^{+} monocytes exit the bone marrow and extravasate into peripheral inflamed tissues, partly in response to chemokines that signal via C-C chemokine receptor type 2 (CCR2) (Serbina and Pamer, 2006; Tsou et al., 2007). They differentiate into inflammatory macrophages and dendritic cells (DCs) that produce tumor necrosis factor (TNF), inducible nitric oxide synthase, and reactive oxygen species in response to bacterial and parasitic infection (Narni-Mancinelli et al., 2011; Robben et al., 2005; Serbina and Pamer, 2006; Serbina et al., 2003b) and can stimulate naive T cells (Geissmann et al., 2003; Serbina et al., 2003a). Ly6C^{+} monocytes are also directly recruited to draining lymph nodes via the high endothelial venules (Palframan et al., 2001). They can produce type 1 interferons in response to viruses via a toll-like receptor 2-dependent pathway (Barbalat et al., 2009). It is also believed that Ly6C^{+} monocytes play a role in chronic inflammation, such as the formation of the atherosclerotic plaque, because *Ccr2*-deficient mice on low density lipoprotein receptor- or apolipoprotein E-deficient backgrounds and a high-fat diet have decreased atherosclerosis (Boring et al., 1998; Dawson et al., 1999).

A second population of blood major histocompatability complex (MHC) class II^{negative} myeloid cells, which lack the Ly6C antigen (and, thus, are termed Ly6C^{low} or Gr1^{low} monocytes), represents a distinct monocyte subset. They develop normally in *Rag*$_2^{-/-}$*Il2rg*$^{-/-}$ mice, which lack lymphoid cells (Auffray et al., 2007). They are characterized by high expression of the C-X3-C chemokine receptor 1 (CX3CR1) and require the transcription factor *Nr4a1* for their development from proliferating bone marrow precursors (Geissmann et al., 2003; Hanna et al., 2011). They crawl along the endothelium of blood vessels in a steady state, express a full set of Fcγ receptors, and mediate IgG-dependent effector functions in mice (Auffray et al., 2007;

 ©2013 Elsevier Inc.

Biburger et al., 2011; Sumagin et al., 2010). These Ly6C^{low} "patrolling" monocytes do not appear to share the functional properties of Ly6C^{+} monocytes. They do not differentiate into inflammatory macrophages or DCs following *Listeria* infection, and their extravasation is a rare event in comparison to Ly6C^{+} monocytes (Auffray et al., 2007). Ly6C^{low} monocytes were suggested to contribute to tissue repair in the myocardium (Nahrendorf et al., 2007), and, in contrast to *Ccr2*-deficient mice, *Nr4a1*-deficient mice showed increased atherosclerosis (Hamers et al., 2012; Hanna et al., 2012). Thus, initial data suggested that Ly6C^{low} monocytes may represent an "anti-inflammatory" subset. However, this hypothesis failed to explain a large number of observations. For example, limiting the recruitment of Ly6C^{low} monocytes after traumatic spinal cord injury was proposed to contribute decreasing inflammation in this model (Donnelly et al., 2011). Several studies on mouse models of lupus nephritis also suggested a proinflammatory role of Ly6C^{low} monocytes, in part via their activation by immune complexes containing nucleic acids (Amano et al., 2005; Santiago-Raber et al., 2009).

Here, we characterize, in several of its key molecular mechanisms, the role of Ly6C^{low} *Nr4a1*-dependent monocytes in vivo as "accessory cells" of the endothelium. Ly6C^{low} monocytes scan capillaries and scavenge micrometric particles from their lumenal side in a steady state. A local nucleic-acid-mediated TLR7 "danger" signal increases their dwell time on the endothelium, a site at which they orchestrate the focal necrosis of endothelial cells that have recruited them, by recruiting neutrophils. TLR7-dependent necrosis is rapid, performed without extravasation, and leaves the basal lamina, tubular epithelium, and glomerular structures intact, at least initially. Phagocytosis of cellular debris suggests that Ly6C^{low} monocytes promote the safe disposal of endothelial cells at the site of recruitment. Therefore, Ly6C^{low} monocytes behave as "housekeepers" of the vasculature, although it is easy to conceive that their action might cause damage itself if the danger signal persists.

RESULTS

CX3CR1^{high} CD11b^{+} Ly6C^{low} Monocytes Are Enriched in the Microvasculature of the Skin and Kidney in a Steady State

Monocytes that adhere to the lumenal side of the endothelium of dermal and heart capillaries, cremaster, mesenteric vessels, and glomeruli in the steady state have been identified by intravital microscopy as CX3CR1^{high} CD11b (αM integrin)$^{+}$ F4/80^{+} leucocytes (Auffray et al., 2007; Hanna et al., 2011; Li et al., 2012; Sumagin et al., 2010; Devi et al., 2013). Crawling CD11b^{+} CX3CR1^{high} monocytes are also present in the vascular network that ramifies around renal tubules in the kidney cortex (Figures 1A and 1B; Movie S1 available online). Analysis of monocyte tethering and adhesion in vivo indicated that crawling Ly6C^{low} monocytes are in constant exchange between the bloodstream and the endothelium, having an average dwell time of 9 min in the kidney microvasculature (Figure 1C; Movies S2 and S3; also see Figure 3). Intravital imaging combined with intravenous (i.v.) immunolabeling of monocytes confirmed that all monocytes that crawled on the endothelium in a steady state expressed CD11b and CX3CR1 and lacked detectable Ly6C staining (Figure 1D; Movies S2, S4, and S5). To investigate the extent of the association of monocytes with the endothelium of the microvasculature in a steady state, we compared the number of monocytes per μl volume in the peripheral blood, the vasculature of the mesentery, and the capillaries of the dermis (ear) and kidney cortex. The number of crawling of Ly6C^{low} CD11b^{+} CX3CR1^{high} monocytes/μl was at least one order of magnitude higher in the dermal and kidney cortex capillaries (10^3 to 10^4 monocytes/μl) than the number of Ly6C^{low} CD11b^{+} CX3CR1^{high} monocytes in the peripheral blood (10^2 monocytes/μl) (Figure 1A). Antibody blockade of αL integrin (CD11a) detached monocytes from the vessel wall in vivo (Auffray et al., 2007), which resulted in a 50% increase in the proportion of circulating Ly6C^{low} over control monocytes (Figure S1), suggesting that the number of cells adherent at any time represent one-third of the total Ly6C^{low} pool that circulate in the peripheral blood.

Crawling CX3CR1^{high} CD11b^{+} Ly6C^{low} Monocytes Survey the Lumenal Side of "Resting" Endothelial Cells and Scavenge Microparticles Attached to It

The characteristic slow motion (10–16 μm/min) and complex tracks, which include U-turns and spirals, of Ly6C^{low} monocytes crawling along the endothelium suggested that they survey the endothelium (Auffray et al., 2007). Intravital microscopy, image deconvolution, and transmission electron microscopy (TEM) indicated that the crawling monocytes extended numerous and mobile filopodia-like structures in contact with the endothelium in the dermal and kidney cortex blood vessels of *Cx3cr1*$^{gfp/+}$*;Rag2*$^{-/-}$*;Il2rg*$^{-/-}$ mice (Figures 1E, 1H, and 1I; Movies S1 and S6). These filopodia or "dendrites" were also observed on human CD14^{dim} monocytes spreading in vitro and stained positively for LFA1 and filamentous actin (Figure S1). Crawling monocytes scavenged 0.2 μm and 2 μm beads that attach to the capillary endothelium in the kidney cortex following i.v. injection, as well as high-molecular-weight dextran (2 MDa; Figures 1F and 1G; Movie S7). Uptake was not followed by their immediate detachment or extravasation. Rather, they can be seen crawling, or patrolling, on the endothelium while carrying their cargo for an extended period of time (e.g., >25 min in Movie S7). Consistently, mononuclear cells with the round or bean-shaped nuclei and granule-poor cytoplasm typical of Ly6C^{low} monocytes (Geissmann et al., 2003) were observed in steady-state kidney capillaries by TEM. These cells were monocytic, not lymphoid, given that they were present in *Rag2*$^{-/-}$*;Il2rg*$^{-/-}$ mice. Pseudopodia that attached to the endothelium, and large endosomes that contained endogenous debris/microparticles were evident (Figures 1H and 1I). Thus, Ly6C^{low} monocytes scan the lumenal side of "resting" endothelial cells and uptake submicrometric and micrometric particles.

LFA1 and ICAM1 and/or ICAM2 Are Absolutely Required for the Crawling of Nr4a1-Dependent MHCII^{neg} Monocytes, but Chemokine Receptors Are Dispensable

Consistent with antibody blockade of LFA1 (Auffray et al., 2007), monocyte attachment to the endothelium was reduced to 1% of wild-type (WT) in *Itgal*$^{-/-}$ mice, whereas monocyte subsets were normally present in the peripheral blood (Figures 2A and 2B). Track analysis of intravital imaging experiments (Figure 2B;

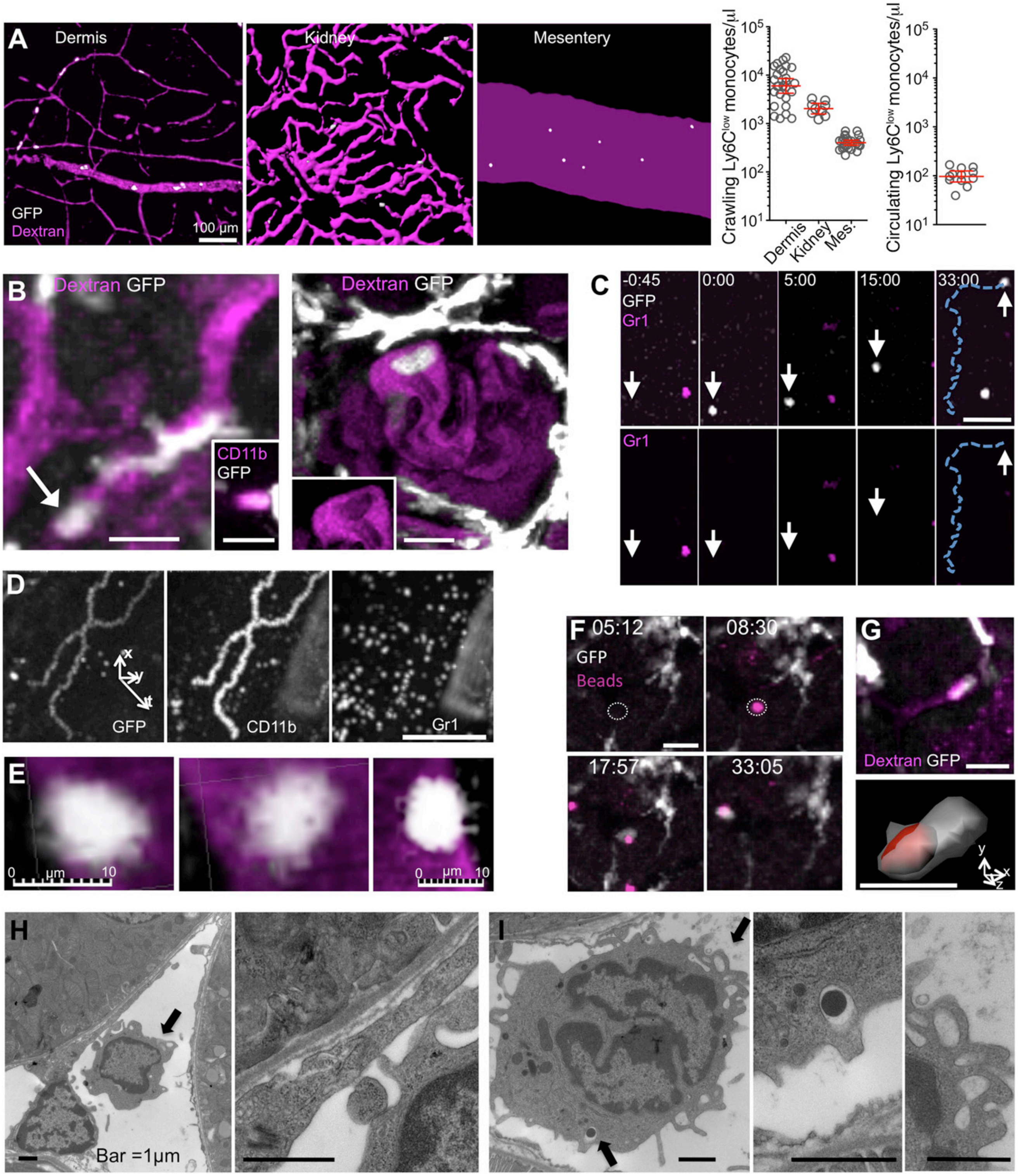

Figure 1. Characterization of Ly6C^{low} Patrolling Monocytes in a Steady State

(A) Left, isovolume-rendered blood vessels (TRITC dextran, magenta) and monocytes (GFP) from the dermis (ear), kidney, and mesentery. The scale bar represents 100 μm. Right, number of crawling CX3CR1^{high} Ly6C^{-} monocytes per μl in the dermal (ear), kidney, and mesentery blood vessels (left) and circulating CX3CR1^{high} Ly6C^{-} monocytes per μl (right). Geometric mean, 95% confidence interval, n $\geq$ 10 fields over $\geq$ 6 mice per condition.

(B) Crawling monocytes (GFP) in a kidney peritubular capillary (left) labeled with CD11b PE Ab (inset) and a glomerulus (right). Capillaries are magenta (TRITC-labeled 70 kD dextran). Shown in the right inset is the TRITC channel alone. The scale bars represent 10 μm.

(legend continued on next page)

Movie S4) comparing $Itgal^{-/-}$ mice and their WT littermates demonstrated that αL integrin was absolutely required for monocyte crawling. The few remaining $Itgal^{-/-}$ monocytes that attached to the endothelium passively followed the blood flow (Figure 2B). LFA1 (αLβ2 integrin) accepts several ligands, including ICAM1, ICAM2, ICAM3, and JAM-A (de Fougerolles et al., 1993; Marlin and Springer, 1987; Ostermann et al., 2002; Staunton et al., 1989). Crawling monocytes were still present in $Icam1^{-/-}$ mice, though they were reduced by 50%, and were normally present in $Icam2^{-/-}$ mice (Figure 2C). However, monocyte attachment to the endothelium was reduced to 2% of control in $Icam1/2^{-/-}$ double mutant mice, and the remaining adherent monocytes passively followed the blood flow, a phenocopy of the $Itgal^{-/-}$ mutant (Figure 2C). Thus, LFA1 and its ligand ICAM1—or ICAM2 in ICAM1-deficient mice—mediate adhesion and crawling of $Ly6C^{low}$ monocytes to the endothelium.

Chemokine receptor *Ccr2* deficiency decreased by half the number of circulating $Ly6C^{+}$ monocytes (Serbina and Pamer, 2006), which are proposed to represent a precursor for $Ly6C^{low}$ monocytes (Varol et al., 2007). In the absence of *Ccr2*, $Ly6C^{+}$ monocytes were decreased by ~50%, as described, but the numbers of $Ly6C^{low}$ monocytes in the bloodstream and crawling on the endothelium were unaffected in comparison to control monocytes (Figures 2D and 2E). *Cx3cr1* deficiency was reported to moderately decrease the numbers of circulating and crawling $Ly6C^{low}$ monocytes (Auffray et al., 2007; Auffray et al., 2009a; Landsman et al., 2009). *Cx3cr1*-deficient crawling monocytes displayed a normal patrolling motility and filopodia formation in vivo (Figure 2F), despite their number being reduced. Therefore, monocyte crawling on the endothelium does not require *Cx3cr1* or *Ccr2*. To test whether another chemokine, or a combination of chemokines, may be responsible for LFA1 activation and binding to ICAM1 and/or ICAM2, we performed intravital imaging experiments in mice after i.v. injection of pertussis toxin (PT), a potent inhibitor of $G\alpha_i$ signaling. PT treatment (up to 100 μg/mouse) did not affect the adhesion and crawling of monocytes on the endothelium (Figure 2G). Thus, it is unlikely that PT-sensitive chemokine receptor signaling controls the adhesion of $Ly6C^{low}$ monocytes to the endothelium in a steady state. A positive control for the effect of PT is shown in Figure 4.

The transcription factor *Nr4a1* is important for the development of $Ly6C^{low}$ monocytes from their bone marrow precursors in mice; circulating and crawling $Ly6C^{low}$ monocytes being reduced by 90% in *Nr4a1*-deficient mice (Hanna et al., 2011). Additional analysis indicated that $CX3CR1^{high}$ $Ly6C^{low}$ $CD11b^{+}$ $I\text{-}A^{-}$ ($MHCII^{-}$) monocytes were, in fact, virtually absent from the blood and from the endothelium of $Nr4a1^{-/-}$ mice (Figures 2H and 2I). The remaining 5%–10% of $Ly6C^{low}$ $CD11b^{+}$ cells in the blood have a distinct phenotype in addition to being *Nr4a1* independent; they express I-A and intermediate levels of CX3CR1 and may represent a previously unrecognized subset of blood myeloid cells independent of both *Ccr2* and *Nr4a1* (Figure 2J, also see Figure S1), which will not be discussed further in this report.

Patrolling Monocytes Are Retained within Kidney Capillaries in TLR7-Mediated Inflammation

Thus, *Nr4a1*-dependent monocytes scavenge the lumenal side of the endothelium in a steady state via a process that requires LFA1 with ICAM1 or ICAM2 interaction but not chemokine-receptor signaling. To evaluate the response of the patrolling monocytes to TLR-mediated signal in vivo, we painted the kidney capsule of $Cx3cr1^{gfp/+}$ mice with R848 (Resiquimod, a selective ligand for TLR7 in mouse), Lipopolysaccharide (LPS), or PBS as a control (Figure S2). After R848 painting, the tracks of crawling monocytes inside capillaries increased in length, and their velocity decreased slightly (Figures 3A and 3B; Movie S8). The duration of their attachment to the endothelium, or dwell time, increased 2- to 3-fold (Figure 3C). This resulted in a rapid, sustained, time- and TLR7-dependent increase in their number within the peritubular capillaries, which was very significantly different from the slight increase observed 3 hr after PBS painting (the latter possibly being due to phototoxicity) (Figure 3D). Retention of crawling monocytes inside capillaries was dependent on local TLR7 signaling, because there was no monocyte retention in $Cx3cr1^{gfp/+};Tlr7^{-/-}$ mice in comparison to $Cx3cr1^{gfp/+};Tlr7^{+/+}$ controls (Figures 3A–3D), although steady-state crawling itself was TLR7-independent (Figures 3A–3D; Movie S8), and because there was no significant monocyte retention in kidney capillaries after i.v. injection of R848 (Figure 3D). In addition, LPS painting did not increase the number of crawling monocytes, in comparison to PBS control (R848-positive control is also shown for clarity; Figure 3D). I.v. injection of labeled antibodies against CD11b 4.5 hr after R848 painting indicated that crawling GFP^{+} $CD11b^{+}$ cells were located inside capillaries (Figure 3E; Movie S9). Moreover, the increase in GFP^{+} cells during the 4.5 hr of the experiment could be wholly accounted for by CD11b-labeled cells, indicating that the crawling monocytes had remained within the vascular lumen (Figure 3F).

(C) CX3CR1-GFP monocytes in a mesenteric blood vessel (top) and Gr1Ab staining (top, bottom); the white arrow follows a $CX3CR1^{+}$ $GR1^{-}$ cell. Time, min:s. The scale bar represents 40 μm.

(D) Fluorescence signal summed over time in the mesenteric blood vessels for CX3CR1-GFP, CD11b Ab, and Gr1Ab. The scale bar represents 100 μm. Data are representative of n = 6 mice.

(E) Deconvolved intravital imaging of CX3CR1-GFP-labeled monocyte in a dermal blood vessel (TRITC-dextran; magenta). Data are representative of >10 mice.

(F) Intravital imaging of 2 μm latex beads (TRITC, magenta) uptake in peritubular capillaries. The bead associates with endothelium (dotted circle) and is phagocytosed by $CX3CR1^{high}$ monocyte. The scale bar represents 20 μm; time, min:s.

(G) Uptake of 2 MDa dextran by a crawling monocyte (GFP) in a kidney peritubular capillary. The bottom shows an isovolume rendering of the same cell. The scale bars represent 10 μm.

(H and I) Representative transmission electron micrograph (TEM) of a mononuclear cell (black arrow) in peritubular capillaries in a $Cx3cr1^{+/gfp};Rag2^{-/-};Il2rg^{-/-}$ mouse. The black arrows in (I) indicate endosomes. The scale bars represent 1 μm.

Also see Figure S1 and Movies S1–S7.

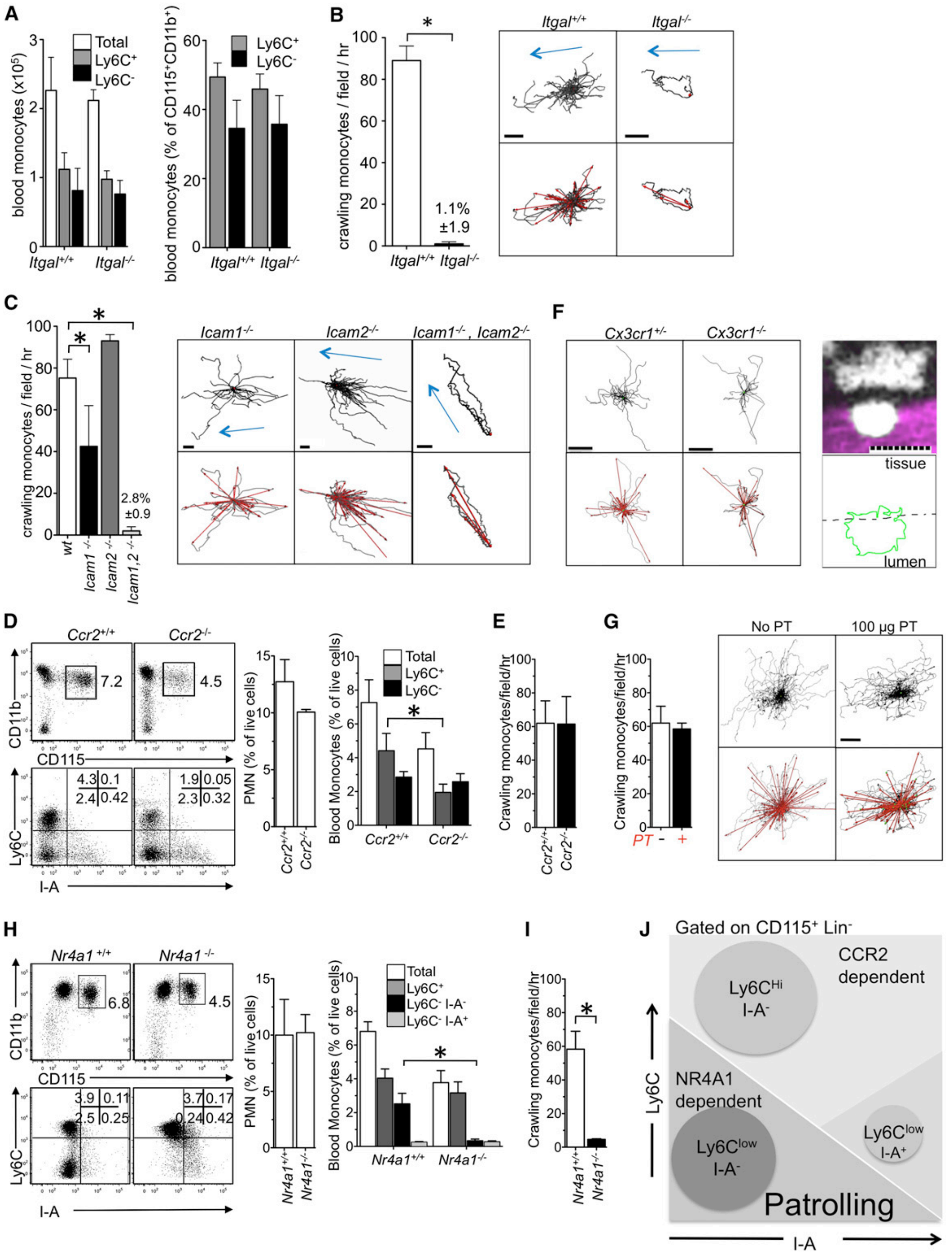

Figure 2. CCR2-Independent, NR4A1-Dependent Ly6C^{low} Monocytes Require LFA1 and ICAM1 or ICAM2, but Not Gαi or CX3CR1, for Intravascular Crawling in a Steady State

(A) Number and percentages of circulating monocyte subsets per ml of blood in *Itgal*$^{+/+}$ and *Itgal*$^{-/-}$ littermates quantified by flow cytometry. Mean ± SEM, n = 3 mice per genotype.

(B) Number and representative tracks and vectors of crawling monocytes per hour per field in the mesenteric blood vessels of *Itgal*$^{+/+}$ and *Itgal*$^{-/-}$ littermates. Mean ± SEM; *, p ≤ 0.05; n = 4 mice per genotype. The scale bars represent 60 μm. Blue arrows indicate blood flow direction.

(C) Data idem as in (B) for *Icam1*$^{-/-}$, *Icam2*$^{-/-}$, and *Icam1*$^{-/-}$ and *Icam2*$^{-/-}$ mice.

(legend continued on next page)

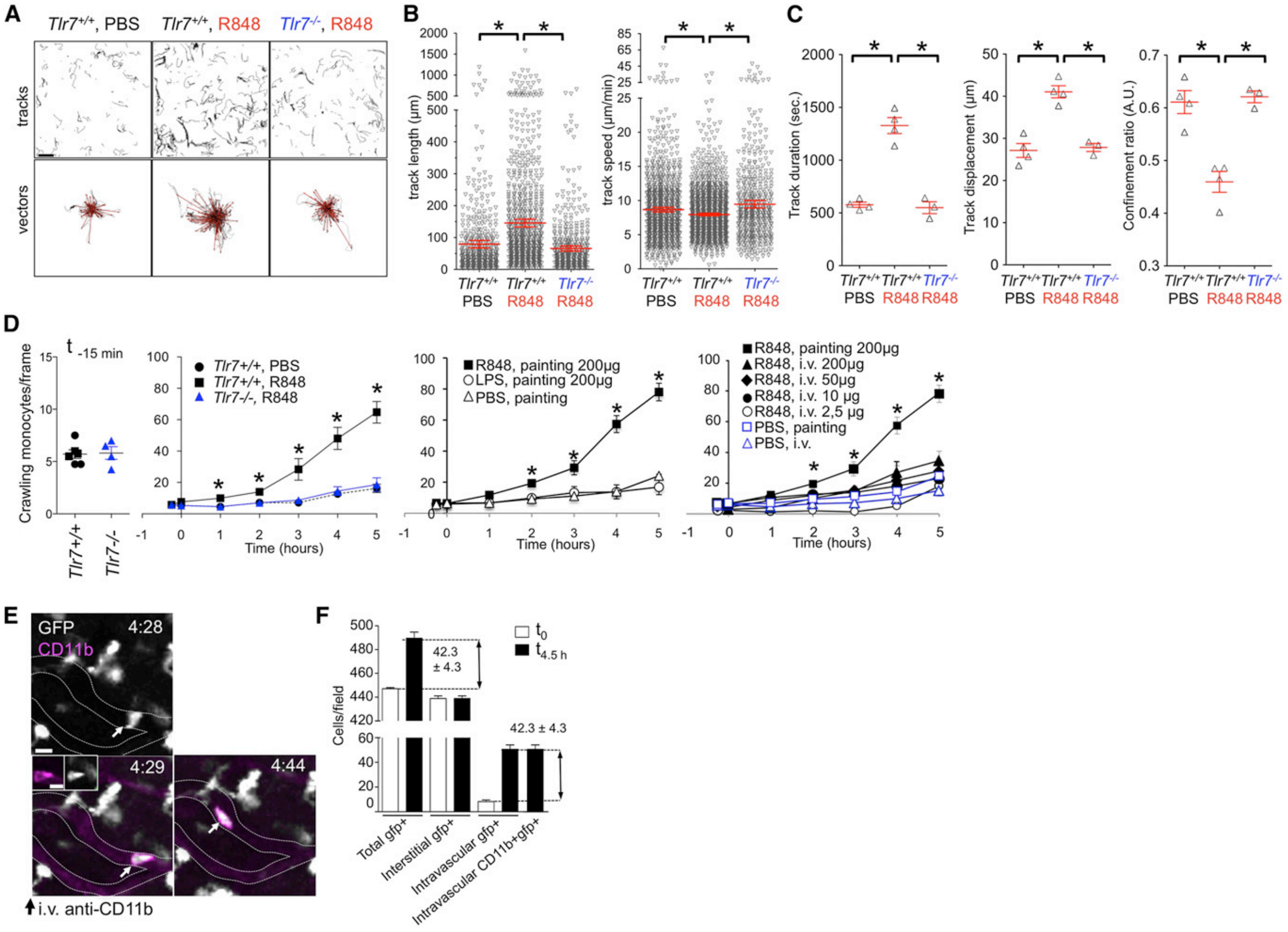

Figure 3. Retention of Crawling Monocytes in the Kidney Vasculature in Response to TLR7 Agonist

(A) Representative monocyte tracks and vectors in the kidney cortex after painting with PBS or R848 in *Tlr7*$^{+/+}$ and *Tlr7*$^{-/-}$ mice over 5 hr. n = 3 or 4 mice per condition The scale bar represents 40 μm.

(B) Track length and speed for monocytes from the experiments described in (A). *, p ≤ 0.05; mean ± SEM.

(C) Mean track duration, track displacement, and confinement ratio of crawling monocytes from the experiments described in (A). *, p ≤ 0.05; mean ± SEM.

(D) Left, cumulative number of crawling monocytes per frame from experiments described in (A). Middle and right, the same experiment split over two graphs for clarity after PBS, LPS, R848 painting, or i.v. injection of PBS or R848. Data points for the R848 painting are shown twice. *, p ≤ 0.05; n = 3-5 mice per condition.

(E and F) Intravital imaging of peritubular capillaries in *Cx3cr1*$^{+/gfp}$ mice after i.v. injection of CD11b-PE (magenta), 4.5 hr after R848 painting, and quantification of GFP^{+} cells in the kidney cortex and capillaries at t0 and 4.5 hr after R848 painting. n = 4, mean ± SEM. The scale bar represents 10 μm.

Also see Figure S2 and Movies S8 and S9.

A Chemokine Receptor Switch Is Responsible for Intravascular Monocyte Retention

These data indicated that crawling monocytes are retained within the capillaries of the kidney cortex in response to a local nucleic acid signal. To eliminate the possibility that lymphoid cells are involved, the experiment was repeated in *Cx3cr1*$^{gfp/+}$*; Rag2*$^{-/-}$*;Il2rg*$^{-/-}$ mice, and the results were identical (Figures 4A and 4B). TLR7 is expressed ubiquitously, including in endothelial cells (Gunzer et al., 2005). After painting with R848, quantitative PCR (qPCR) analysis indicated that the expression of

(D and E) Circulating and crawling monocyte subsets and PMNs in *Ccr2*$^{+/+}$ and *Ccr2*$^{-/-}$ mice. *, p ≤ 0.05; mean ± SEM; n = 3 mice per genotype.

(F) Representative tracks, vectors, and confocal micrograph of crawling monocytes in mesenteric blood vessels of *Cx3cr*$^{-/+}$ and *Cx3cr1*$^{-/-}$ mice (white, CX3CR1-GFP; magenta, TRITC-70kD dextran). The scale bars represent 10 μm. n = 5 mice.

(G) Data idem as in (B) for mice treated with pertussis toxin (PT) 100 μg i.v. Mean ± SEM, n = 2 mice per condition.

(H and I) Data idem as in (D) for *Nr4a1*$^{+/+}$ and *Nr4a1*$^{-/-}$ mice. Mean ± SEM, n = 6 mice per genotype.

(J) Schematic representation of the monocyte subsets. The x axis represents I-A expression, and the y axis represents Ly6C expression divided by *Nr4a1* and *Ccr2* requirement.

Also see Figure S1 and Movie S4.

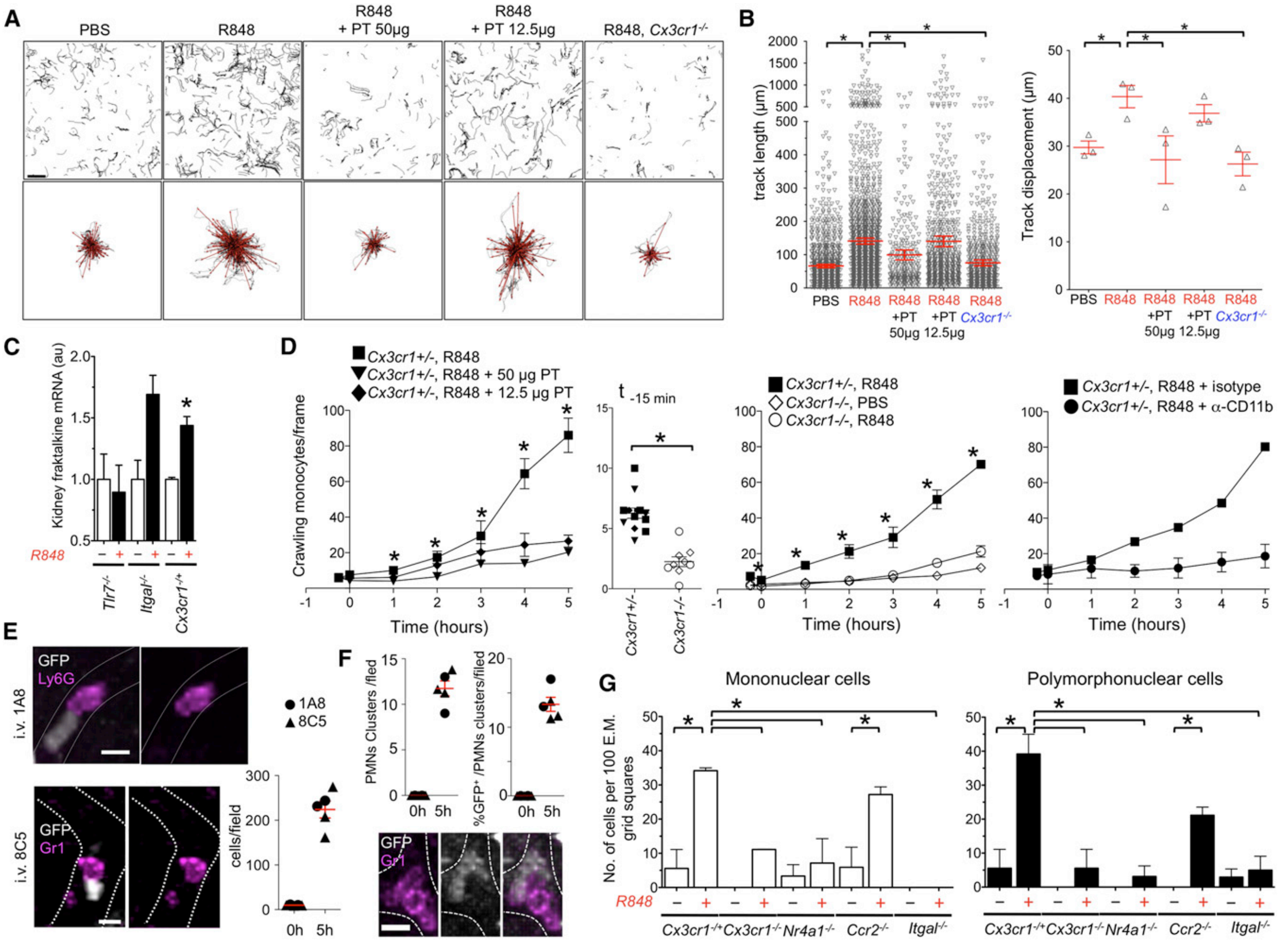

Figure 4. Retention of Crawling Monocytes in the Kidney Vasculature in Response to TLR7 Agonist Requires Chemokine Receptor Signaling

(A) Experiments performed idem as in Figure 3 for *Cx3cr1*$^{+/-}$ *Rag2*$^{-/-}$ *Il2rg*$^{-/-}$ or *Cx3cr1*$^{-/-}$ *Rag2*$^{-/-}$ *Il2rg*$^{-/-}$ mice. The scale bar represents 40 μm. n = 3 mice per condition.

(B) Data idem as in Figures 3B and 3C. *, $p \leq 0.05$; mean ± SEM; n = 3 mice.

(C) qPCR for fractalkine (*Cx3cl1*) messenger RNA in kidney cortex tissue from mice with the indicated genotypes 5hr after painting with R848 or PBS (n = 3–5 mice). *,$p \leq 0.05$; mean ± SEM.

(D) Data idem as in Figure 3D for the stated genotypes and conditions. Right, mice received control or blocking anti-CD11b Ab i.v. injection immediately prior to the experiment. *, $p \leq 0.05$; mean ± SEM; n = 3–5 mice per condition.

(E) Intravital imaging of peritubular capillaries in *Cx3cr1*$^{+/gfp}$ mice 5 hr after R848 painting and immediately after i.v. injection of 10 μg Gr1-APC or Ly6G-PE antibodies. Mean ± SEM, n = 4 mice per condition.

(F) GFP^{-} Gr1^{+} and Ly6G^{+} cells forming clusters in capillaries before and after R848 painting and proportion of clusters in contact with a GFP^{+} cells. n = 5 mice per condition, mean ± SEM.

(G) TEM of the superficial kidney cortex from mice 5 hr after PBS or R848 painting. Cells/100 TEM grid squares; mean ± SEM; n = 4 *Cx3cr1*$^{+/-}$ mice per condition, 2 *Cx3cr1*$^{-/-}$ mice per condition, 2 *Nr4a1*$^{-/-}$ mice per condition, 2 *Ccr2*$^{-/-}$ mice per condition, and 3 *Itgal*$^{-/-}$ mice per condition. *, $p < 0.05$.

Also see Figure S3 and Movie S10.

fractalkine (CX3CL1) in the kidney cortex is rapidly upregulated in a TLR7-dependent manner and independently of leucocyte adhesion (Figure 4C). I.v. injection of PT inhibited, in a dose-dependent manner, the increase in track length and displacement in response to R848 painting and the resulting accumulation of monocytes inside kidney capillaries (Figures 4A, 4B, and 4D). Thus, fractalkine was upregulated in the kidney, and Gα_i chemokine-receptor signaling was required to retain monocytes in the capillaries by preventing their detachment from the endothelium. One obvious candidate to mediate this effect was the fractalkine receptor CX3CR1. Indeed, *Cx$_3$cr1* deficiency prevented monocyte retention inside kidney capillaries in response to R848 (Figures 4A–4D). In a steady state, crawling monocytes are present, though they are less abundant in the vasculature of *Cx$_3$cr1*$^{-/-}$ mice (Auffray et al., 2007) (Figures 4D). In addition, Mac1 (αMβ2 integrin) blockade with neutralizing antibodies, which does not affect "steady-state" crawling behavior (Auffray et al., 2007) (Figure 4D), also prevented the accumulation of monocytes inside kidney capillaries (Figure 4D). Therefore, although Gα_i signaling is dispensable for monocyte adhesion in

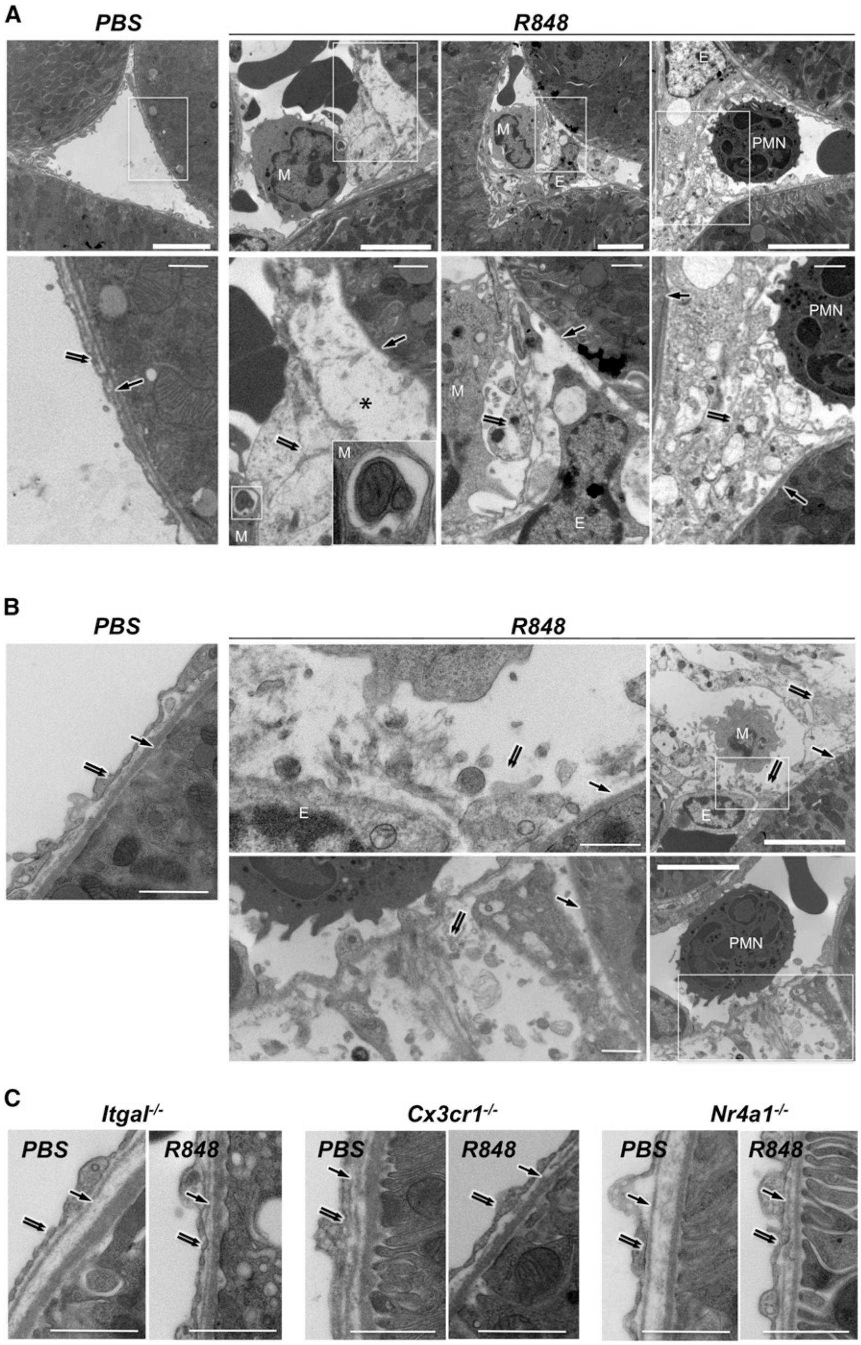

Figure 5. Focal Necrosis of Endothelial Cells

(A) Representative electron micrographs of kidney cortex peritubular capillaries 5 hr after painting with R848 or PBS from Figure 4G. Single arrow, basal lamina; double arrow, endothelium; *, fluid in the subendothelial space; M, mononuclear cell; PMN, polymorphonuclear cell; E, endothelial cell nucleus. In the PBS-treated healthy peritubular capillary, endothelial cells are flat and close to basal lamina. The R848-treated peritubular capillary shows a swollen necrotic endothelial cell, expanded subendothelial space, mononuclear cell phagocytosing mitochondria, and blebbing necrotic endothelium (images are from *Cx3cr*$^{-/+}$ *Rag2*$^{-/-}$*IL2rg*$^{-/-}$ mice).

(B) Similar features to those represented in (A) are shown for *Ccr2*$^{-/-}$ mice.

(C) Representative healthy endothelium in R848- and PBS-treated kidneys from *Itgal*$^{-/-}$, *Cx3cr1*$^{-/-}$, and *Nr4a1*$^{-/-}$ mice. Micrographs are representative of experiments analyzed in Figure 4G. The thick scale represents 5 μm, the thin scale represents 1 μm. Initial magnification was 15,000×.

Also see Figure S3.

a steady state, it is required in response to R848 in order to prevent the detachment of crawling monocytes and promote their intravascular retention, at least in part via fractalkine and CX3CR1 and αM integrin.

Intravascular Retention of Monocytes Is CCR2 Independent and Causes Neutrophil Recruitment

Although we did not reproducibly detect crawling granulocytes in the kidney capillaries of WT mice in a steady state by intravital microscopy or TEM, the above experiments documented the recruitment of GFP^{-} Gr1^{+} Ly6G^{+} cells, most likely to be neutrophilic granulocytes, crawling inside capillaries and forming clusters in the vicinity of the patrolling monocytes (Figures 4E and 4F; Movie S10). TEM analysis of the kidney cortex of mice 5 hr after painting with R848 confirmed the recruitment of both monocytic cells and granulocytes in the vasculature (Figure 4G). Monocytes and neutrophils were attached to the endothelium of peritubular and glomerular capillaries (Figures 5 and S3). However, we did not observe any example of monocyte or neutrophil diapedesis or the presence of neutrophils outside the capillaries. These results are consistent with data obtained by intravital microscopy. They also indicated that leucocytes were retained not only in peritubular but also in glomerular capillaries (Figure S3). Mice were not submitted to intravital microscopy in these TEM experiments; thus, leucocyte recruitment was independent from laser damage. Similar observations were made in *Ccr2*-deficient mice (Figure 4G), indicating that CCR2 is largely dispensable for the retention of crawling monocytes and the recruitment of neutrophils. However, both monocyte and neutrophil recruitment were severely decreased in *Itgal*-, *Cx3cr1*-, and *Nr4a1*-deficient mice (Figure 4G). Given that neutrophils do not express CX3CR1 and are present in normal numbers in *Nr4a1*-deficient mice, these data provided genetic evidence suggesting that monocytes recruit neutrophils after their retention in the microvasculature of the kidney.

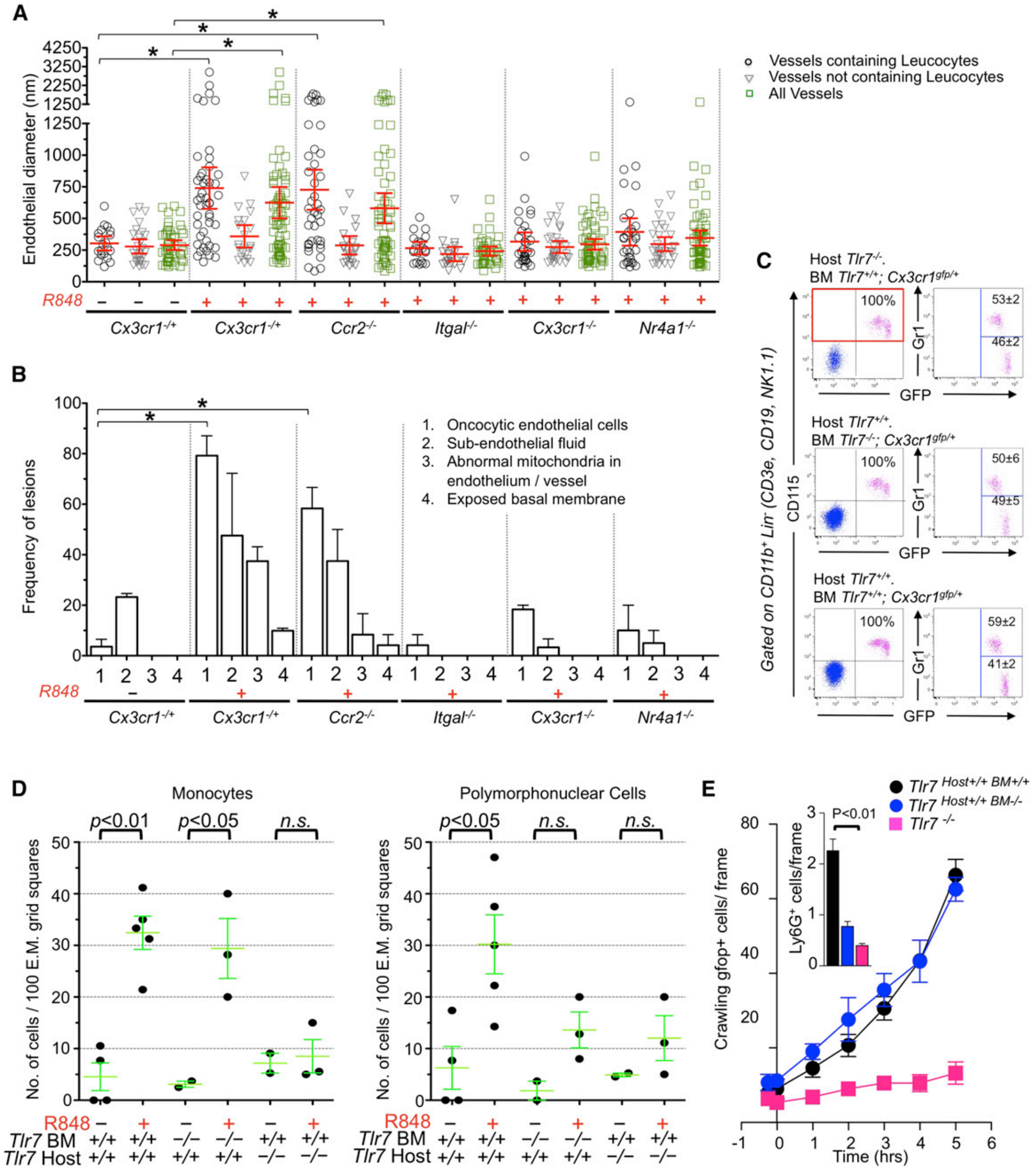

Figure 6. Focal Endothelial Necrosis Requires Retention of Ly6C^{low} Monocytes, which Requires Expression of TLR7 on the Kidney

(A and B) Endothelial thickness measurements and microscopic features from the TEM results described in Figure 4G. At least 30 capillaries were examined per condition. Mean ± 95% confidence interval (A) or SEM (B).*, $p < 0.05$.

(C) Representative FACS dot plots from the blood of bone marrow chimera. Mean ± SEM, n = 5–8 mice per condition.

(D) Intravascular mononuclear cells (left) and polymorphonuclear cells (right) quantified by TEM in bone marrow chimera. Cell counts are from individual mice. Mean and SEM, n = 2–5 mice.

(E) Intravascular monocytes (left) and granulocytes (right) quantified by intravital microscopy in the kidney cortex of bone marrow chimera following R848 painting. Granulocytes ($\times 10^2$) were quantified at 5 hr immediately after i.v. injection of the Ly6G antibody. Mean ± SEM, n = 3 or 4 mice per condition.

Also see Figure S3.

Intravascular Monocytes Orchestrate the Rapid Necrosis and Disposal of Endothelial Cells

TEM indicated that the endothelium of the tubular capillaries was undergoing severe focal damage at sites where monocytes, and neutrophils were retained after TLR7 stimulation. Endothelium thickness was increased (Figures 5 and 6A), and endothelial cells were markedly swollen with rarefaction of the cytoplasm, blebbing from the plasma membrane of cytoplasmic fragments, loss of plasma membrane integrity, and release of cellular debris and damaged organelles, such as mitochondria, whereas the

morphology of nuclei remained largely unchanged (Figures 5 and 6B). In addition, extracellular fluids accumulated in the subendothelial space, separating the endothelial cells from the basal lamina (Figure 6B). In some cases, endothelial cells were detached from the basal lamina and a monocyte was seen in contact with the basal lamina (Figures 6B and S3). Endothelial cell damage was limited to cells adjacent to a monocyte or a neutrophil, and the basal lamina was always preserved (Figure 5A). Monocytes adjacent to the damaged endothelial cells could be observed phagocytosing cellular debris and organelles such as altered mitochondria (Figures 5A and S3). These features corresponded to a "textbook" description of necrosis and also suggested a safe disposal of the endothelial cells debris and organelles by monocytes. Similar features were observed in *Ccr2*-deficient mice (Figures 5B, 6A, and 6B). In contrast, endothelial damage was absent in *Itgal*-, *Cx3cr1*-, and *Nr4a1*-deficient mice after kidney painting with either PBS or R848 (Figures 5C, 6A, and 6B). Therefore, focal necrosis of endothelial cells and phagocytosis of cellular debris required the presence of leucocytes on the endothelium and was *Cx3cr1*- and *Nr4a1*-dependent but largely *Ccr2*-independent. Altogether, these data indicated that patrolling *Nr4a1*-dependent monocytes orchestrate and are required for endothelial cell death and scavenge the resulting cellular debris in situ.

The Kidney Endothelium Retains Monocytes, which, In Turn, Recruit Neutrophils that Kill Endothelial Cells

We investigated the signals responsible for monocyte and neutrophil recruitment by TEM and intravital analysis of TLR7-deficient bone marrow chimeric mice (Figure 6C). Expression of TLR7 on the host, but not on monocytes, was required for their recruitment in the kidney vasculature (Figures 6D and 6E). This indicated that the kidney endothelium recruits monocytes in response to a nucleic acid signal sensed via TLR7, consistent with fractalkine induction by R848 and fractalkine- and CX3CR1-dependent recruitment of monocytes (see Figure 4). However, the efficient recruitment of neutrophils required TLR7 expression on both the host and bone-marrow-derived cells (Figures 6D and 6E). Expression of TLR7 by the kidney and the retention of TLR7-deficient monocytes by the endothelium were not sufficient to recruit neutrophils. These data characterize a sequence of events and the successive requirement of TLR7 on the kidney for the accumulation of monocytes on the endothelium and on hematopoietic cells for the recruitment of neutrophils.

Endothelial cell necrosis was reduced to background levels in *Tlr7*$^{host+/+BM-/-}$ despite the presence of monocytes (Figure 7A), suggesting either that monocytes require TLR7 to kill endothelial cells or that neutrophils are responsible for endothelial necrosis. Therefore, we selectively depleted neutrophils (by 90%) but not monocytes by intraperitoneal injection of an antibody against Ly6G 1A8 8 hr before R848 painting (Figure 7B). Neutrophil depletion from the periphery resulted in the severe reduction of neutrophils in the kidney, whereas monocytes were still retained (Figure 7C), and mostly abolished endothelial necrosis (Figures 7D and 7E). Therefore, the endothelium recruits monocytes, monocytes recruit neutrophils, and the neutrophils are, in turn, required for endothelial killing.

Consistent with a role of monocytes in recruiting neutrophils in a TLR7-dependent manner, fluorescence-activated cell sorting (FACS)-sorted Ly6C^{low} monocytes displayed a strong MEK-dependent proinflammatory chemokine and cytokine response to R848 in vitro, characterized by the production of the chemokine KC (C-X-C chemokine ligand 1; CXCL1), known to contribute to neutrophil recruitment, as well as several other proinflammatory mediators such as interleukin 1β (IL-1β), TNF, C-C cheomokine ligand 3 (CCL3; macrophage inflammatory protein 1α), and interleukin 6 (IL-6) (Figure 7F). Notably, this response appears to be relatively specific, or at least preferential, for TLR7, given that Ly6C^{low} monocytes responded very poorly to LPS stimulation both in vitro and in vivo (Figure S2), which is in contrast to Ly6C^{+} monocytes (Figure 7F) and consistent with data in humans (Cros et al., 2010).

DISCUSSION

A Multistep Process Controls Intravascular Scavenging of the Endothelium and Removal of Endothelial Cells

Our data indicate that intravascular patrolling, mediated by LFA1-ICAM1 interactions and independent of chemokine signaling, represents the first step of monocyte surveillance of the endothelium from its lumenal side. TLR7-dependent sensing of a "danger" signal by the kidney cortex then triggers the expression of fractalkine and intravascular retention of Ly6C^{low} monocytes by the endothelium. This process is Gαi-dependent and requires the fractalkine receptor CX3CR1 expressed by Ly6C^{low} monocytes and the αMβ2 integrin Mac1 (Figure 7G). The subsequent recruitment of neutrophils requires the prior retention of Ly6C^{low} monocytes and the expression of TLR7 by hematopoietic cells. Altogether, our data suggest that the activation of intravascular monocytes via TLR7 in prolonged contact with the endothelium is the mechanism that recruits neutrophils via the production of KC or other proinflammatory mediators. In the last steps, neutrophils, in turn, mediate the focal necrosis of the endothelial cells, and monocytes scavenge cellular debris, all from within the capillary lumen. Phagocytosis of cellular debris suggests the safe disposal of endothelial cells at the site of necrosis. Therefore, Lyc6C^{low} monocytes behave as "housekeepers" of the vasculature.

Earlier observations that Ly6C^{low} monocytes crawl on endothelia (Auffray et al., 2007; Hanna et al., 2011; Li et al., 2012; Sumagin et al., 2010) and do not contribute to the pool of inflammatory monocytes that extravasate to give inflammatory macrophages and DCs in response to listeria infection in vivo (Auffray et al., 2007; Geissmann et al., 2003; Serbina et al., 2003b) are consistent with their intravascular function. Their MEK-dependent preferential response to TLR7 agonists is reminiscent of our earlier observation that CD14^{dim} human monocytes selectively respond to viruses and nucleic acids via a TLR7-8 MEK pathway (Cros et al., 2010) and further suggests that Ly6C^{low} and CD14^{dim} monocytes share a common function in mice and humans, respectively.

Neutrophils damage endothelial cells when activated (Villanueva et al., 2011; Westlin and Gimbrone, 1993). There has been recent recognition that apoptosis was not the only mechanism underlying programmed or regulated cell death and that

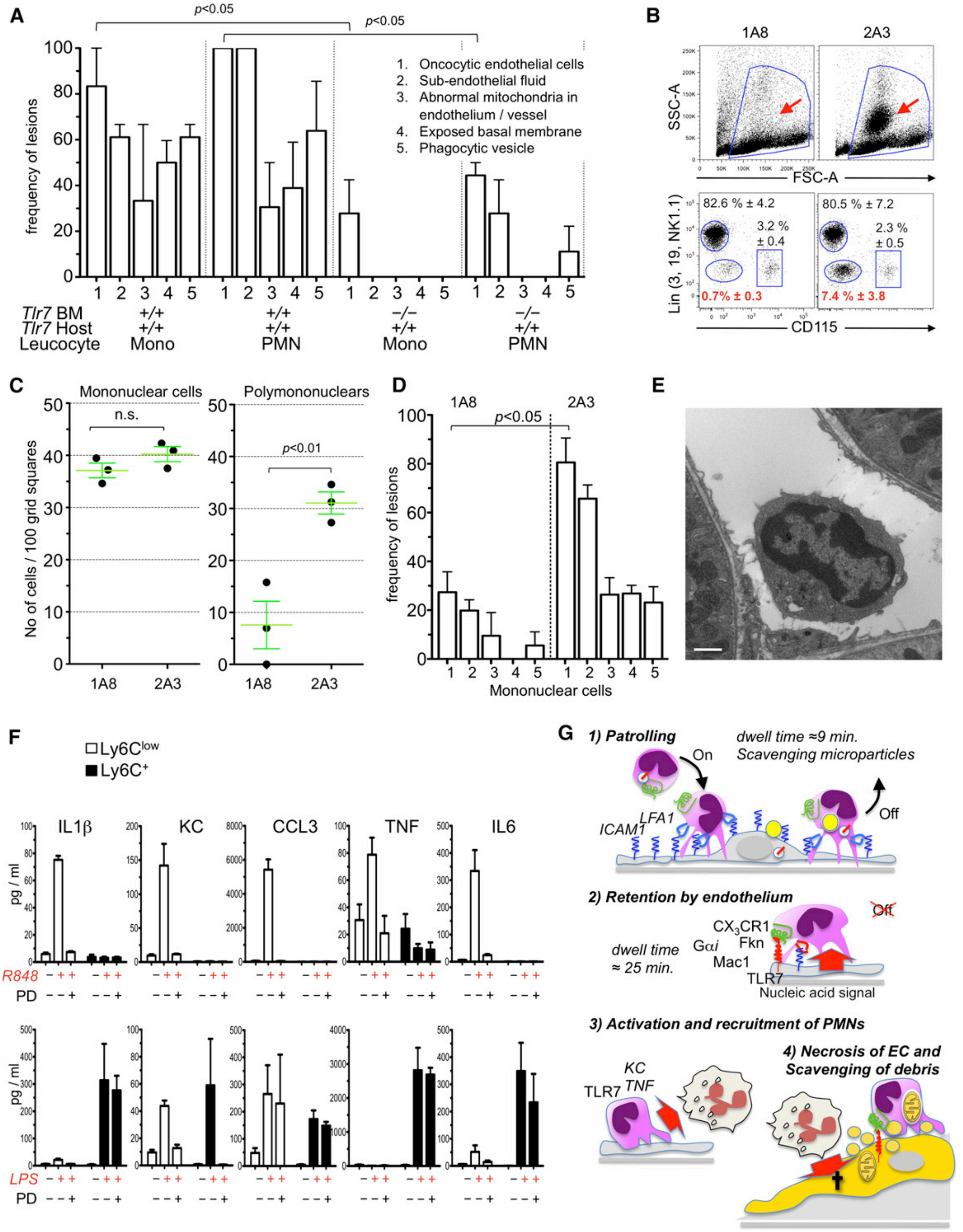

Figure 7. Neutrophils Kill Endothelial Cells

(A) Endothelial cell microscopic features of chimeric mice described in Figure 6D expressed as the percentage of mononuclear cells (mono) or PMN-containing fields that present with the indicated lesions.

(B) Representative FACS dot plots of peripheral blood cells of mice treated 8 hr earlier with Ly6G-depleting Ab (1A8) or isotype control (2A3). The arrow in the FSC/SSC panel indicates granulocytic cells, the percentage of Lin^{-} $CD115^{-}$ granulocytes are indicated in red. n = 3 mice per group, mean ± SEM.

(C) Presence of intravascular mononuclear (left) and polymorphonuclear cells (right) as quantified by TEM in mice treated with 1A8 or 2A3 8 hr before kidney painting with R848. n = 3 mice per group, mean ± SEM.

(D) Endothelial cell microscopic features of granulocyte-depleted and control mice. n = 3 mice per group, mean ± SEM.

(E) Representative peritubular capillary containing a monocyte from a 1A8-treated mouse.

(legend continued on next page)

necrotic cell death can occur in vivo (Edinger and Thompson, 2004; Galluzzi and Kroemer, 2008; Green, 2011; Kroemer et al., 2009). Indeed, our data demonstrate that neutrophils can mediate endothelial cell death by necrosis in vivo. Activated neutrophils produce a variety of soluble and membrane-bound mediators that can contribute to necrosis, and additional investigation should explore the exact mechanisms responsible for neutrophil-mediated necrosis of endothelial cells.

Possible Relevance to Vascular Inflammation and Tissue Damage

The several steps that allow Ly6C^{low} monocytes to orchestrate endothelial cell death indicate a tight control of endothelial cell necrosis, which may be useful in avoiding excessive damage. However, as outlined above, it is easy to conceive that this process might become detrimental, particularly if the danger signal persists in situations such as atherosclerosis or systemic lupus erythematosus (SLE). For example, TLR7 is involved in several steps of the pathogenesis of SLE (Barrat et al., 2007; Deane et al., 2007; Vollmer et al., 2005), and subendothelial deposits of nucleic acids in immune complexes are a feature of a proportion of SLE patients (Hill et al., 2001; Hill et al., 2000). Activation of Ly6C^{low} monocytes and their human equivalent was reported in murine models of SLE and human patients (Amano et al., 2005; Nakatani et al., 2010; Santiago-Raber et al., 2009; Cros et al., 2010; Yoshimoto et al., 2007), and CX3CR1 blockade was proposed to reduce monocyte recruitment to the kidney and inflammation (Inoue et al., 2005; Nakatani et al., 2010). Collectively, this literature raises the possibility that, although Ly6C^{low} monocytes would be expected to protect the endothelium, they could also paradoxically contribute to vascular and tissue damage in genetically susceptible individuals.

Revising the Leucocyte Diapedesis Model

Extravasation of leucocytes into inflamed tissues by the means of chemotaxis is a hallmark of inflammation, and it is unclear why monocytes and neutrophils did not extravasate in response to the local TLR7-mediated signal. It is possible that additional signals are needed. However, the accumulation of crawling leucocytes inside blood vessels may not always lead to extravasation (Geissmann et al., 2005; Devi et al., 2013). Metchnikoff (1893)'s description of diapedesis 120 years ago in his ninth lecture on the comparative pathology of inflammation insisted that the accumulation and ameboid locomotion of leucocytes inside blood vessels was not always followed by extravasation. Intravascular leucocytes retained both ameboid motility and chemotaxis, and Metchnikoff (1893) proposed that they sensed and obeyed signals from the inflamed tissues to stay inside blood vessels, a process called "negative chemotactism." Whether nucleic acids represent such a negative chemotactic factor in vivo is an interesting hypothesis that would have practical implications. The "choice" between extravasation and intravascular "retention" may also correspond to distinct properties of different leucocyte cell types. It is clear from the present study that the Ly6C^{low} subset of monocytes specializes in surveying the endothelium. Therefore, we suggest that interactions between leucocyte and endothelium may be best described by a revised model that takes into account subset-specific functions, time, and the response to individual stress signals, as opposed to the leucocyte extravasation model alone.

EXPERIMENTAL PROCEDURES

Mice

Mouse strains are described in Extended Experimental Procedures.

Antibodies and Reagents

Antibody clones and reagent manufacturers are described in Extended Experimental Procedures.

Intravital Microscopy and Image Analysis of the Ear, Mesentery, and Kidney

Intravital confocal microscopy of monocytes in the ear and mesentery was performed as previously described (Auffray et al., 2007) with LSM510 Zeiss and SP5 Leica inverted microscopes. For intravital imaging of the kidney, we induced anaesthesia with a combination of ketamine, xylazine, and acepromazine, and the kidney was surgically exposed without removing the renal capsule or interrupting the blood flow and placed against a coverslip. Anesthesia was maintained by the inhalation of isoflurane in oxygen, and the animal was imaged for up to 5 hr (see Extended Experimental Procedures). Cells in blood vessels were tracked and analyzed as described in Extended Experimental Procedures.

Transmission Electron Microscopy

The full methods for TEM are described in Extended Experimental Procedures. In brief, kidneys were prepared as for intravital imaging but not illuminated. Instead, after 5 hr, the animal was euthanized and the kidney tissue was fixed in 2.5% gluteraldehyde overnight at 4°C. Samples were processed and sectioned to reveal superficial peritubular capillaries and glomeruli. Mononuclear and polymorhonuclear cells were counted for each grid square imaged. Endothelial thickness was measured from the outer edge of the nearest basal lamina to the lumen of the vessel to the outer edge of the lumenal side of the endothelial cell. We were careful to measure equivalent areas in all vessels. Oncocytic endothelial cells and the related features of subendothelial swelling, basal membrane exposure, mitochondrial abnormality, and phagocytosis were quantified and normalized per image and leukocyte.

Statistical Tests

In the figures, the asterisk represents $p \leq 0.05$ in an unpaired Student's t test. Otherwise, p values from unpaired Student's t test are indicated.

Flow Cytometry

Flow cytometry was performed as described in Extended Experimental Procedures.

Multiplexed ELISA for In Vitro Cytokine Production

Multiplexed ELISA for in vitro cytokine production was performed as described in Extended Experimental Procedures.

(F) Proinflammatory cytokine production in vitro by sorted Ly6C^{low} and Ly6C^{+} monocytes after 24 hr stimulation with medium alone or R848 (top) in the absence or presence of a MEK inhibitor (PD) or for medium alone or LPS (bottom) in the absence or presence of the MEK inhibitor (PD) (bottom). Multiplexed ELISA, n = 3 mice per condition.
(G) Schematic representation of the molecular and cellular features of the interaction of Ly6C^{low} monocytes with the endothelium in a steady state and TLR7-mediated endothelial "safe disposal."
Also see Figure S3.

Animal Experiments

Animal experiments were performed in strict adherence to our United Kingdom Home Office project license issued under the Animals (Scientific Procedures) Act 1986.

SUPPLEMENTAL INFORMATION

Supplemental Information includes Extended Experimental Procedures, three figures, and ten movies and can be found with this article online at http://dx.doi.org/10.1016/j.cell.2013.03.010.

ACKNOWLEDGMENTS

I. Charo, B. Engelhardt and J.V. Stein, and R. Alon kindly provided *Ccr2*$^{-/-}$, *Icam1*$^{-/-}$ and *Icam2*$^{-/-}$, and *Igal*$^{-/-}$ mice, respectively. The authors are also indebted to G. Burn and A. Fischer for helpful discussions, to C. Trouillet for the management of the Geissmann lab, and to A. McGuigan and her colleagues from the Biological Service Unit at King's College London, Guy's Campus, for support with mouse husbandry. E.G.S. is supported by a PhD fellowship from the Oliver Bird Rheumatism Programme, Nuffield Foundation, UK. Work was supported by grants to F.G. from the Medical Research Council, UK (MRCG0900867), the European Research Council (ERC-2010-StG-261299 MPS2010), and the European Science Foundation (ERC EURYI Award).

Received: September 21, 2012
Revised: January 14, 2013
Accepted: March 5, 2013
Published: April 11, 2013

REFERENCES

Amano, H., Amano, E., Santiago-Raber, M.L., Moll, T., Martinez-Soria, E., Fossati-Jimack, L., Iwamoto, M., Rozzo, S.J., Kotzin, B.L., and Izui, S. (2005). Selective expansion of a monocyte subset expressing the CD11c dendritic cell marker in the Yaa model of systemic lupus erythematosus. Arthritis Rheum. *52*, 2790–2798.

Auffray, C., Fogg, D., Garfa, M., Elain, G., Join-Lambert, O., Kayal, S., Sarnacki, S., Cumano, A., Lauvau, G., and Geissmann, F. (2007). Monitoring of blood vessels and tissues by a population of monocytes with patrolling behavior. Science *317*, 666–670.

Auffray, C., Fogg, D.K., Narni-Mancinelli, E., Senechal, B., Trouillet, C., Saederup, N., Leemput, J., Bigot, K., Campisi, L., Abitbol, M., et al. (2009a). CX3CR1+ CD115+ CD135+ common macrophage/DC precursors and the role of CX3CR1 in their response to inflammation. J. Exp. Med. *206*, 595–606.

Auffray, C., Sieweke, M.H., and Geissmann, F. (2009b). Blood monocytes: development, heterogeneity, and relationship with dendritic cells. Annu. Rev. Immunol. *27*, 669–692.

Barbalat, R., Lau, L., Locksley, R.M., and Barton, G.M. (2009). Toll-like receptor 2 on inflammatory monocytes induces type I interferon in response to viral but not bacterial ligands. Nat. Immunol. *10*, 1200–1207.

Barrat, F.J., Meeker, T., Chan, J.H., Guiducci, C., and Coffman, R.L. (2007). Treatment of lupus-prone mice with a dual inhibitor of TLR7 and TLR9 leads to reduction of autoantibody production and amelioration of disease symptoms. Eur. J. Immunol. *37*, 3582–3586.

Biburger, M., Aschermann, S., Schwab, I., Lux, A., Albert, H., Danzer, H., Woigk, M., Dudziak, D., and Nimmerjahn, F. (2011). Monocyte subsets responsible for immunoglobulin G-dependent effector functions in vivo. Immunity *35*, 932–944.

Boring, L., Gosling, J., Cleary, M., and Charo, I.F. (1998). Decreased lesion formation in CCR2-/- mice reveals a role for chemokines in the initiation of atherosclerosis. Nature *394*, 894–897.

Cros, J., Cagnard, N., Woollard, K., Patey, N., Zhang, S.Y., Senechal, B., Puel, A., Biswas, S.K., Moshous, D., Picard, C., et al. (2010). Human CD14dim monocytes patrol and sense nucleic acids and viruses via TLR7 and TLR8 receptors. Immunity *33*, 375–386.

Dawson, T.C., Kuziel, W.A., Osahar, T.A., and Maeda, N. (1999). Absence of CC chemokine receptor-2 reduces atherosclerosis in apolipoprotein E-deficient mice. Atherosclerosis *143*, 205–211.

de Fougerolles, A.R., Klickstein, L.B., and Springer, T.A. (1993). Cloning and expression of intercellular adhesion molecule 3 reveals strong homology to other immunoglobulin family counter-receptors for lymphocyte function-associated antigen 1. J. Exp. Med. *177*, 1187–1192.

Deane, J.A., Pisitkun, P., Barrett, R.S., Feigenbaum, L., Town, T., Ward, J.M., Flavell, R.A., and Bolland, S. (2007). Control of toll-like receptor 7 expression is essential to restrict autoimmunity and dendritic cell proliferation. Immunity *27*, 801–810.

Devi, S., Li, A., Westhorpe, C.L., Lo, C.Y., Abeynaike, L.D., Snelgrove, S.L., Hall, P., Ooi, J.D., Sobey, C.G., Kitching, A.R., and Hickey, M.J. (2013). Multiphoton imaging reveals a new leukocyte recruitment paradigm in the glomerulus. Nat. Med. *19*, 107–112.

Donnelly, D.J., Longbrake, E.E., Shawler, T.M., Kigerl, K.A., Lai, W., Tovar, C.A., Ransohoff, R.M., and Popovich, P.G. (2011). Deficient CX3CR1 signaling promotes recovery after mouse spinal cord injury by limiting the recruitment and activation of Ly6Clo/iNOS+ macrophages. J. Neurosci. *31*, 9910–9922.

Edinger, A.L., and Thompson, C.B. (2004). Death by design: apoptosis, necrosis and autophagy. Curr. Opin. Cell Biol. *16*, 663–669.

Galluzzi, L., and Kroemer, G. (2008). Necroptosis: a specialized pathway of programmed necrosis. Cell *135*, 1161–1163.

Geissmann, F., Jung, S., and Littman, D.R. (2003). Blood monocytes consist of two principal subsets with distinct migratory properties. Immunity *19*, 71–82.

Geissmann, F., Cameron, T.O., Sidobre, S., Manlongat, N., Kronenberg, M., Briskin, M.J., Dustin, M.L., and Littman, D.R. (2005). Intravascular immune surveillance by CXCR6+ NKT cells patrolling liver sinusoids. PLoS Biol. *3*, e113.

Green, D.R. (2011). The end and after: how dying cells impact the living organism. Immunity *35*, 441–444.

Gunzer, M., Riemann, H., Basoglu, Y., Hillmer, A., Weishaupt, C., Balkow, S., Benninghoff, B., Ernst, B., Steinert, M., Scholzen, T., et al. (2005). Systemic administration of a TLR7 ligand leads to transient immune incompetence due to peripheral-blood leukocyte depletion. Blood *106*, 2424–2432.

Hamers, A.A., Vos, M., Rassam, F., Marinković, G., Kurakula, K., van Gorp, P.J., de Winther, M.P., Gijbels, M.J., de Waard, V., and de Vries, C.J. (2012). Bone marrow-specific deficiency of nuclear receptor Nur77 enhances atherosclerosis. Circ. Res. *110*, 428–438.

Hanna, R.N., Carlin, L.M., Hubbeling, H.G., Nackiewicz, D., Green, A.M., Punt, J.A., Geissmann, F., and Hedrick, C.C. (2011). The transcription factor NR4A1 (Nur77) controls bone marrow differentiation and the survival of Ly6C- monocytes. Nat. Immunol. *12*, 778–785.

Hanna, R.N., Shaked, I., Hubbeling, H.G., Punt, J.A., Wu, R., Herrley, E., Zaugg, C., Pei, H., Geissmann, F., Ley, K., and Hedrick, C.C. (2012). NR4A1 (Nur77) deletion polarizes macrophages toward an inflammatory phenotype and increases atherosclerosis. Circ. Res. *110*, 416–427.

Hill, G.S., Delahousse, M., Nochy, D., Tomkiewicz, E., Rémy, P., Mignon, F., and Méry, J.P. (2000). A new morphologic index for the evaluation of renal biopsies in lupus nephritis. Kidney Int. *58*, 1160–1173.

Hill, G.S., Delahousse, M., Nochy, D., Rémy, P., Mignon, F., Méry, J.P., and Bariéty, J. (2001). Predictive power of the second renal biopsy in lupus nephritis: significance of macrophages. Kidney Int. *59*, 304–316.

Inoue, A., Hasegawa, H., Kohno, M., Ito, M.R., Terada, M., Imai, T., Yoshie, O., Nose, M., and Fujita, S. (2005). Antagonist of fractalkine (CX3CL1) delays the initiation and ameliorates the progression of lupus nephritis in MRL/lpr mice. Arthritis Rheum. *52*, 1522–1533.

Kroemer, G., Galluzzi, L., Vandenabeele, P., Abrams, J., Alnemri, E.S., Baehrecke, E.H., Blagosklonny, M.V., El-Deiry, W.S., Golstein, P., Green, D.R., et al.; Nomenclature Committee on Cell Death 2009. (2009). Classification of cell death: recommendations of the Nomenclature Committee on Cell Death 2009. Cell Death Differ. *16*, 3–11.

Landsman, L., Bar-On, L., Zernecke, A., Kim, K.W., Krauthgamer, R., Shagdarsuren, E., Lira, S.A., Weissman, I.L., Weber, C., and Jung, S. (2009). CX3CR1 is required for monocyte homeostasis and atherogenesis by promoting cell survival. Blood *113*, 963–972.

Li, W., Nava, R.G., Bribriesco, A.C., Zinselmeyer, B.H., Spahn, J.H., Gelman, A.E., Krupnick, A.S., Miller, M.J., and Kreisel, D. (2012). Intravital 2-photon imaging of leukocyte trafficking in beating heart. J. Clin. Invest. *122*, 2499–2508.

Marlin, S.D., and Springer, T.A. (1987). Purified intercellular adhesion molecule-1 (ICAM-1) is a ligand for lymphocyte function-associated antigen 1 (LFA-1). Cell *51*, 813–819.

Metchnikoff, E. (1893). Lecture IX. Lectures on the Comparative Pathology of Inflammation Delivered at the Pasteur Institute in 1891 (London: Kegan Paul, Trench, Trubner & CO), pp. 144–147.

Nahrendorf, M., Swirski, F.K., Aikawa, E., Stangenberg, L., Wurdinger, T., Figueiredo, J.L., Libby, P., Weissleder, R., and Pittet, M.J. (2007). The healing myocardium sequentially mobilizes two monocyte subsets with divergent and complementary functions. J. Exp. Med. *204*, 3037–3047.

Nakatani, K., Yoshimoto, S., Iwano, M., Asai, O., Samejima, K., Sakan, H., Terada, M., Hasegawa, H., Nose, M., and Saito, Y. (2010). Fractalkine expression and CD16+ monocyte accumulation in glomerular lesions: association with their severity and diversity in lupus models. Am. J. Physiol. Renal Physiol. *299*, F207–F216.

Narni-Mancinelli, E., Soudja, S.M., Crozat, K., Dalod, M., Gounon, P., Geissmann, F., and Lauvau, G. (2011). Inflammatory monocytes and neutrophils are licensed to kill during memory responses in vivo. PLoS Pathog. *7*, e1002457.

Ostermann, G., Weber, K.S., Zernecke, A., Schröder, A., and Weber, C. (2002). JAM-1 is a ligand of the beta(2) integrin LFA-1 involved in transendothelial migration of leukocytes. Nat. Immunol. *3*, 151–158.

Palframan, R.T., Jung, S., Cheng, G., Weninger, W., Luo, Y., Dorf, M., Littman, D.R., Rollins, B.J., Zweerink, H., Rot, A., and von Andrian, U.H. (2001). Inflammatory chemokine transport and presentation in HEV: a remote control mechanism for monocyte recruitment to lymph nodes in inflamed tissues. J. Exp. Med. *194*, 1361–1373.

Robben, P.M., LaRegina, M., Kuziel, W.A., and Sibley, L.D. (2005). Recruitment of Gr-1+ monocytes is essential for control of acute toxoplasmosis. J. Exp. Med. *201*, 1761–1769.

Santiago-Raber, M.L., Amano, H., Amano, E., Baudino, L., Otani, M., Lin, Q., Nimmerjahn, F., Verbeek, J.S., Ravetch, J.V., Takasaki, Y., et al. (2009). Fcgamma receptor-dependent expansion of a hyperactive monocyte subset in lupus-prone mice. Arthritis Rheum. *60*, 2408–2417.

Serbina, N.V., and Pamer, E.G. (2006). Monocyte emigration from bone marrow during bacterial infection requires signals mediated by chemokine receptor CCR2. Nat. Immunol. *7*, 311–317.

Serbina, N.V., Kuziel, W., Flavell, R., Akira, S., Rollins, B., and Pamer, E.G. (2003a). Sequential MyD88-independent and -dependent activation of innate immune responses to intracellular bacterial infection. Immunity *19*, 891–901.

Serbina, N.V., Salazar-Mather, T.P., Biron, C.A., Kuziel, W.A., and Pamer, E.G. (2003b). TNF/iNOS-producing dendritic cells mediate innate immune defense against bacterial infection. Immunity *19*, 59–70.

Serbina, N.V., Jia, T., Hohl, T.M., and Pamer, E.G. (2008). Monocyte-mediated defense against microbial pathogens. Annu. Rev. Immunol. *26*, 421–452.

Staunton, D.E., Dustin, M.L., and Springer, T.A. (1989). Functional cloning of ICAM-2, a cell adhesion ligand for LFA-1 homologous to ICAM-1. Nature *339*, 61–64.

Sumagin, R., Prizant, H., Lomakina, E., Waugh, R.E., and Sarelius, I.H. (2010). LFA-1 and Mac-1 define characteristically different intralumenal crawling and emigration patterns for monocytes and neutrophils in situ. J. Immunol. *185*, 7057–7066.

Tsou, C.L., Peters, W., Si, Y., Slaymaker, S., Aslanian, A.M., Weisberg, S.P., Mack, M., and Charo, I.F. (2007). Critical roles for CCR2 and MCP-3 in monocyte mobilization from bone marrow and recruitment to inflammatory sites. J. Clin. Invest. *117*, 902–909.

Varol, C., Landsman, L., Fogg, D.K., Greenshtein, L., Gildor, B., Margalit, R., Kalchenko, V., Geissmann, F., and Jung, S. (2007). Monocytes give rise to mucosal, but not splenic, conventional dendritic cells. J. Exp. Med. *204*, 171–180.

Villanueva, E., Yalavarthi, S., Berthier, C.C., Hodgin, J.B., Khandpur, R., Lin, A.M., Rubin, C.J., Zhao, W., Olsen, S.H., Klinker, M., et al. (2011). Netting neutrophils induce endothelial damage, infiltrate tissues, and expose immunostimulatory molecules in systemic lupus erythematosus. J. Immunol. *187*, 538–552.

Vollmer, J., Tluk, S., Schmitz, C., Hamm, S., Jurk, M., Forsbach, A., Akira, S., Kelly, K.M., Reeves, W.H., Bauer, S., and Krieg, A.M. (2005). Immune stimulation mediated by autoantigen binding sites within small nuclear RNAs involves Toll-like receptors 7 and 8. J. Exp. Med. *202*, 1575–1585.

Westlin, W.F., and Gimbrone, M.A., Jr. (1993). Neutrophil-mediated damage to human vascular endothelium. Role of cytokine activation. Am. J. Pathol. *142*, 117–128.

Yoshimoto, S., Nakatani, K., Iwano, M., Asai, O., Samejima, K., Sakan, H., Terada, M., Harada, K., Akai, Y., Shiiki, H., et al. (2007). Elevated levels of fractalkine expression and accumulation of CD16+ monocytes in glomeruli of active lupus nephritis. Am. J. Kidney Dis. *50*, 47–58.

EXTENDED EXPERIMENTAL PROCEDURES

Mice

Cx3cr1$^{gfp/+}$, triple mutant CX3CR1 competent Cx3cr1$^{gfp/+}$, Rag2$^{-/-}$, IL2rg$^{-/-}$ mice and CX3CR1 deficient Cx3cr1$^{gfp/gfp}$, Rag2$^{-/-}$, IL2rg$^{-/-}$ mice, devoid of all lymphoid cells and in which monocytes are the only GFP-expressing cells, have been described previously (Auffray et al., 2007; Geissmann et al., 2003). C57BL/6 (B6) mice were generated in-house or purchased from Harlan Laboratories or Charles River UK. Cx3cr1$^{gfp/gfp}$, Rag2$^{-/-}$, IL2rg$^{-/-}$ mice were bred with B6 mice to produce Cx3cr1$^{gfp/+}$, Rag2$^{+/-}$, IL2rg$^{+/-}$ mice. Tlr7 null mice (on C57BL/6 background) were previously reported (Hemmi et al., 2002). Female B6 or Tlr7$^{-/-}$ mice were crossed with male Cx3cr1$^{gfp/gfp}$, Rag2$^{-/-}$, IL2rg$^{-/-}$ mice to generate male Cx3cr1$^{gfp/+}$, Rag2$^{+/-}$, IL2rg$^{+/+}$, Tlr7$^{+/+}$ or Cx3cr1$^{gfp/+}$, Rag2$^{+/-}$, IL2rg$^{+/+}$, Tlr7$^{-/-}$ mice respectively. Nr4a1$^{-/-}$ (B6.129S2-Nr4a1tm1Jmi/J) mice (Lee et al., 1995), lacking in Ly6C^{low} monocytes (Hanna et al., 2011) were purchased from Jackson Laboratories as frozen embryos, rederived in-house and bred from heterozygotes to provide knockout and wild-type littermate controls. Itgal$^{-/-}$ mice, deficient for alpha-L integrin (CD11a)(Ding et al., 1999), crossed with UBC-EGFP mice (Schaefer et al., 2001) (Jackson Laboratories), were a kind gift from Ronen Alon (Weizmann Institute of Science, Israel) and were bred from heterozygotes to provide knockout and wild-type littermate controls. Icam1$^{-/-}$,2$^{-/-}$ double knockout mice (Boscacci et al., 2010) deficient in both ICAM1 (CD54) and ICAM2 (CD102) were a kind gift from Britta Engelhardt and Jen Stein (University of Bern, Switzerland). They were rederived in house as Icam1$^{-/+}$,2$^{-/+}$ and bred to produce double and single knockouts and knockout and wild-type littermate controls.

Solutions and Buffers

Phosphate Buffered Saline (-)Ca, (-)Mg (PBS; Life Technologies). PBS containing 1% Bovine Serum Albumin (w/v) (Life Technologies) and 0.1% Tween20 (v/v)(Sigma) is referred to as PBS-T. Hank's balanced salt solution (HBSS; Life Technologies) was supplemented with 20mM HEPES (Sigma). Opti-MEM serum free growth media (Life Technologies). Mouse RBC lysis buffer contained 8.3g NH_4Cl 1g $NaHCO_3$ 1mL EDTA (100mM) in 1L ddH2O. PBS containing 0.5% (w/v) BSA and 2mM EDTA (PBS-BSA-EDTA). Electron microscopy (EM) fixative was 2.5% EM grade gluteraldehyde in 0.1 M phosphate buffer pH7.3. Tris-EDTA (TE).

Antibodies and Reagents

Anti-human CD11a (Clone 38; Autogen Bioclear), DAPI (Invitrogen), Phalloidin AlexaFluor488 (Invitrogen), Vectashield Hard Set mounting medium with Prolong Gold Anti-fade (Vector Laboratories). Anti-mouse CD11a (M17/4; BD Parmingen). Rat anti-mouse CD11b PE, NA/LE, PE Cy7 or AlexaFluor647 (M1/70; BD Pharmingen), Rat IgG2b isotype control (A95-1; BD Pharmingen), Anti-mouse Gr1APC (Ly6C/Ly6G; RB6-8C5 BD Pharmingen), Anti-mouse Ly6G (1A8; Bio X Cell). Rat IgG2a isotype control (2A3; Bio X Cell or BD PharMingen) anti-mouse Ly6G PE (1A8; BD Pharmingen), Anti-mouse CD115 FITC (CSF1R; AFS98 BD PharMingen), Anti-mouse CD16/32 (2.4G2BD Pharmingen), Anti-mouse CD3 Biotin (145 2C-11; BD PharMingen), Anti-mouse NK1.1Biotin (PK136; BD Pharmingen), Anti-mouse CD19 Biotin (1D3; BD PharMingen). Streptavadin Pacific Blue (Invitrogen). Anti-mouse I-A(b) PE (AF6 120.1 BD Pharmingen) Anti-mouse I-A/E FITC (2G9 BD Pharmingen). Anti-mouse LAMP1 (Rabbit polyclonal; Sigma). DyLight 549 goat anti-mouse IgG2a (Jackson Immuno Research). MEK inhibitor PD98059 (PD; Enzo Lifescience), R848 (InvivoGEN) and LPS from E. Coli 0111:B4 (#L4391; Sigma). Pertussis Toxin (PT; Tocris #3097). R848 was reconstituted at 1 mg/ml with sterile water. LPS was reconstituted at 1 mg/ml with sterile PBS.

Generation of Bone Marrow Chimeras

Bone marrow (BM) recipient 6-week-old male C57BL/6 or *Tlr7*$^{-/-}$ mice were exposed to a single lethal dose of 10Gy total body irradiation. Irradiated mice were allowed to rest for 3 hr. Sex and age matched donor *Tlr7*$^{+/+}$ (*Cx3cr1*$^{gfp/+}$, *Rag2*$^{+/-}$, *Il2rg*$^{+/+}$, *Tlr7*$^{+/+}$) or *Tlr7*$^{-/-}$ (*Cx3cr1*$^{gfp/+}$, *Rag2*$^{+/-}$, *Il2rg*$^{+/+}$, *Tlr7*$^{-/-}$) mice were sacrificed and BM cells were harvested in RPMI (Sigma; supplemented with 1% Penicillin/Streptomycin). 3x10^{7} BM cells (in PBS) were injected iv via the tail vein into the congenic irradiated mice. Chimerism was assessed by flow cytometry after 6 weeks of BM reconstitution and mice were used for experiments 7 weeks after BM reconstitution.

In Vivo Depletion of Neutrophils

7-8 weeks-old C57BL/6 mice were injected ip with 10mg/Kg of body weight neutrophil-depleting anti-Ly6G (clone 1A8; Bio X Cell, West Lebanon, NH, US) or isotype control antibody (clone 2A3; Bio X Cell). After 8 hr, neutrophil depletion was assesses by flow cytometry and the mice were subjected to kidney painting with R848 as below.

Intravital Microscopy and Image Analysis of the Ear, Mesentery, and Kidney

Microscopy

Intravital confocal microscopy of monocytes in the ear and mesentery was performed similarly to previously described (Auffray et al., 2007). Briefly, mice were anesthetized using a cocktail of ketamine (50 mg/kg), xylazine (10 mg/kg), and acepromazine (1.7 mg/kg) injected intraperitoneally and were kept at 37°C and received oxygen (0.5 l/min). Anesthesia was maintained by half-dose boosts delivered subcutaneously every 30 min (ear and mesentery model) or by continuous inhalation of 0.5% isoflurane in oxygen (Merial, Harlow, United Kingdom) in the kidney model. The mouse was positioned on a custom made aluminum tray stage insert with circular

2.5 cm diameter hole, covered with a glass coverslip attached with silicone grease. Images were acquired using either an inverted Leica TCS SP5 DMI6000 confocal laser scanning system using Argon-ion 488nm, DPSS 561nm, HeNe 633nm laser lines through 10 × 0.4 N.A. HCX PL APO and 20x 0.5 N.A PL Fluotar air objectives or an inverted Zeiss LSM 510 confocal laser scanning system equipped with 10 × /0.5 Fluar and 20x/0.75 Plan Apochromat objectives. A thermostat controlled heated chamber (Life Imaging Services) was used to keep the whole microscope, mice, tray, and microscope objectives at 37°C during the experiment.

Dermal Blood Vessels

Mice were anesthetized and the inside of the unshaved ear was placed in a drop of PBS directly against the coverslip and held in position with tape over a second small square coverslip to hold it in place. 80 μl of TRITC conjugated 70kDa dextran (70 μM) was injected intravenously if using *Cx3cr1*$^{gfp/+}$, *Rag2*$^{-/-}$, *IL2rg*$^{-/-}$ or *Cx3cr1*$^{gfp/gfp}$, *Rag2*$^{-/-}$, *IL2rg*$^{-/-}$ mice. In other mice, 10 μg PE-conjugated anti-CD11b (M1/70) or APC anti-Gr1 (Rb6-8c5) was injected intravenously, to stain the CD11b+ and Gr1+ cells and circulating unbound antibody revealed the blood vessels. Imaging was performed as described previously using the Zeiss LSM510 (Auffray et al., 2007), or using 10 × 0.4 N.A. HCX PL APO and 20 x 0.5 N.A PL Fluotar air objectives on the Leica SP5 with 488 and 561nm laser lines for GFP and PE respectively. Emission wavelengths were selected using the spectral scanning head to exclude cross channel bleed-through.

Mesenteric Blood Vessels

Mice were anesthetized and the skin and peritoneum were carefully cut and the longest portion of the intestine (proximal to the colon) was placed on the coverslip. To avoid perturbation by intestinal peristalsis, the intestine was immobilized using small sheets of paper. Vessel walls of the branches of the mesenteric vein and mesenteric artery were detected by bright-field transmitted light imaging. Images were acquired as described (Auffray et al., 2007), or using 10 × 0.4 N.A. HCX PL APO and 20 x 0.5 N.A PL Fluotar air objectives on the Leica SP5 with 488 and 561 nm laser lines for GFP and PE respectively. For intravital phenotyping experiments, anti-CD11b PE (10 μg) and/or anti-Gr1APC (10 μg) was injected intravenously. For steady-state intravital Gαi blocking experiments 100μg pertussis toxin (PT) was injected intravenously and the mice were imaged 2 hr later.

Kidney Peritubular Capillaries

Mice were anesthetized and the fur from the left flank region was removed using a hair trimmer. The left kidney was surgically exposed without removing the renal capsule or interrupting the blood flow, and placed on the coverslip on PBS-soaked strips of paper. The animal was further stabilized on the stage by two strips of tape applied gently over the front and back legs of the mouse. In order to visualize the blood vessels 120 μl of TRITC conjugated 70kDa (70 μM) or 2 MDa (2 μM) dextran was injected intravenously. Images were acquired using 20x 0.5 NA PL Fluotar air and 40x 1.25 NA APO CS oil objectives on the Leica SP5 with 488, 561 and 633nm laser lines for GFP, PE and APC respectively. For intravital phenotyping experiments, anti-CD11b PE (10 μg), anti-Gr1APC (10 μg) or anti-Ly6G PE (10 μg) was injected intravenously. For beads-scavenging experiments, 20 μl 2 μm TRITC-labeled beads were injected intravenously 5 min after starting imaging.

Direct Treatment of Kidney with R848

The kidney was initially preimaged for 15 min. Then, (t = 0), 400 μl R848 or LPS 0.5 mg/ml was applied over the exteriorized kidney in order to induce kidney inflammation, and the animal was imaged for further 5 hr. As a control, the kidney was painted with PBS alone. For intravital Gαi blocking experiments 50 or 12.5 μg (dosage used stated in figure) PT was injected intravenously at t = 0 simultaneously with R848 kidney painting and the kidney was imaged for further 5 hr as previously. For intravital CD11b (Mac-1) blocking experiments 4 mg/kg anti-CD11b NA/LE or Rat IgG2b isotype control was injected intravenously at t = 0 simultaneously with R848 kidney painting and the kidney was imaged for further 5 hr as previously.

Analysis of the Number of Crawling Monocytes per μl Volume

Blood vessels were fitted with "isovolumes" using Imaris software (Bitplane) to calculate the imaged blood vessel volume. The sum of the fitted volume was calculated and the number of crawling monocytes within this volume quantified at 4-5 different time points. The number of crawling monocytes per time point was then was then divided by the volume calculated in μl.

Cell Track Analysis

To produce summed fluorescence images over time from differentially labeled cells, time-lapse z-stacks were individually maximally projected, then complete track paths were then generated by maximally projecting each snapshot of the time-series into a single image as described previously (Auffray et al., 2007). To compare crawling monocyte paths in different knockout animals and in in vivo blocking experiments in the mesenteric vessels, cells were tracked using the autoregressive-motion algorithm and filtered for a minimum track length of 30 μm from their origin and a minimum track duration of 3 min, then were manually assessed and edited for track continuity. Motile tissue GFP^{+} cells were excluded by masking. For the kidney, the numerous GFP^{+} cells made manual tracking of each individual monocyte necessary. In the kidney capillaries each cell was manually tracked (by marking its position in each snapshot of the time-series), accurate z positioning was achieved using a software function that automatically positions the point at the center-of-mass of the fluorescent signal in z. For each field, tracks were 'translated' to a common origin in space to allow direct comparison (number, direction and displacement). Tracks were then quantified for number, speed, length, duration, displacement and confinement ratio (defined as the quotient of track length and track displacement).

Transmission Electron Microscopy

Mice were anesthetized and the left kidney was exteriorized as before. Without any illumination or imaging, the animal was placed on a Petri dish, and transferred to a temperature controlled recovery chamber set at 37°C. The kidney was painted with R848 or PBS as a

control. After 5 hr, the animal was euthanized and the kidney removed. The kidney was halved to just leave the half that was lying in the R848 solution, the tissue was sliced into approximately 1mm thick slices and fixed in 2.5% gluteradehyde (v/v) in 0.1M phosphate buffer (pH 7.3) overnight at 4°C. Subsequently samples were washed several times in phosphate buffer and postfixed in 1% osmium tetroxide in 0.1M phosphate buffer pH7.3 for 1.5 hr at 4°C. Samples were then washed, dehydrated in a graded series of ethanol and equilibrated with propylene oxide before infiltration with TAAB epoxy resin (TAAB Laboratories Equipment, Reading, UK). Tissue slices were cut into smaller pieces just before embedding and polymerized at 70°C for 24 hr. Ultrathin sections (70-90 nm) were prepared to reveal superficial peritubular capillaries and also slightly deeper to reveal glomeruli using a Reichert-Jung Ultracut E ultramicrotome (Leica), then mounted on 150 mesh copper grids, contrasted using uranyl acetate and lead citrate. Samples were imaged using a CCD camera (Hamamatsu) contained in a digital imaging system (AMT) and H7600 transmission electron microscope (Hitachi) at 75kV.

TEM Quantification

Mononuclear and polymorphonuclear cells were counted for each EM grid square imaged. Endothelial thickness was measured from the outer edge of the nearest basal membrane to the lumen of the vessel to the outer edge of the lumenal side of the endothelial cell. Care was taken to measure equivalent areas in all vessels. Endothelial cell nuclei and the corners of vessels were not measured. 5-10 measurements were taken for 10 randomly selected vessels per grid and additionally categorized for the presence or absence of leukocytes in the blood vessel. Oncoytic endothelial cells and the related features of endothelial / sub-endothelial swelling, basal membrane exposure and externalized or abnormal mitochondria and phagocytic vesicles were counted and the number of such lesions where normalized to the number of images or images containing leukocytes for each experimental condition as noted in figure legends.

qPCR of Murine Renal Tissue

Kidneys were treated with R848, LPS or PBS as a control in vivo as above for 5 hr without imaging. After removal of the kidney capsule, approx. 5mg of kidney tissue (a 1mm x 1mm block) from the cortex was taken and frozen using liquid nitrogen immediately in a nucleic acid and RNase free microcentrifuge tube. Tissue was stored at −80°C until it was homogenized in RLT plus buffer (QIAGEN) containing 1% 2-mercaptoethanol (Sigma-Aldrich) by drawing through a 21 g hypodermic needle > 15 times. Subsequently total RNA was purified using an RNeasy plus micro kit (QIAGEN), according to manufacturer's instructions. RNA was quantified by absorbance spectroscopy (Nanodrop) and reverse transcription was performed using Superscript III reverse transcriptase and first strand buffer (Invitrogen) and random hexamer primers (Fermantas) according to manufacturer's instructions (with the addition of RNasin; Promega). qPCR analysis was performed by the SYBRgreen method using a Rotorgene qPCR machine (QIAGEN) and SensiMix SYBR noROX qPCR mastermix reagents (Bioline) using the following primers: Gapdh, for 5′ATTGTGGAAGGGC TCATGACC3′ rev 5′TCTTCTGGGTGGCAGTGATG3′; 18 s, for 5′AACGGCTACCACATCCAAGG3′ rev 5′GGGAGTGGGTAATT TGCGC3′; *Cx3cl1 (fractalkine)*, 5′GCGTGCCATTGTCCTGGAGACG3′ rev 5′TTCGGGTCAGCACAGAAGCGT3′; *IL1b*, for 5′TGAAAGACGGCACACCCACCC3′ rev. 5′TTGCTTGGGATCCACACTCTCCA 3′ (Sigma); *Tnf*, Mm_Tnf_1_SG QuantiTect Primer Assay QT00104006 (QIAGEN). mRNA was quantified using the standard curve method, samples were normalized using *18 s* or *Gapdh* mRNA concentration and expressed as fold change over control PBS treated kidney tissue.

Statistical Tests

Where * is used in the figures, $p \leq 0.05$ in an unpaired Student's t test otherwise the *P* value is given in the figure. N for the experiment is given in the figure legend. Tests were performed using GraphPad Prism (GraphPad).

Flow Cytometry

Flow Cytometry to Phenotype Mouse Blood Monocytes

Red blood cell lysis was performed using mouse RBC lysis buffer (see recipe above) on 200-900 μl whole blood collected from the tail vein or by cardiac puncture. ~2-12x10^6 leukocytes were resuspended in PBS-BSA 0.5% (w/v) and blocked with anti-mouse CD16/32 for 10 min on ice, then stained with anti-CD3, NK1.1, CD19 biotin (Lineage Stain; Lin^-), anti-CD115 FITC, CD11b PE Cy7, I-A(b) PE, Gr1APC and Strepavadin Pacific Blue, or anti-CD3, NK1.1, CD19 biotin (Lineage Stain; Lin^-), anti-CD115 PE, CD11b PE Cy7, I-A FITC, Gr1APC and Strepavadin Pacific Blue. Samples were analyzed on a Aria II custom FACS (BD) with 405, 488, 561 and 633 nm laser lines. Lin^- cells were analyzed for CD11b, CD115, Gr1 and I-A expression. In order to analyze blood monocytes after R848 kidney painting, the following protocol was applied. The mice were anesthetized and the left kidney was exteriorized as previously. The animal was put on a petri dish, and then transferred to a temperature controlled recovery chamber set at 37°C. The kidney was painted with R848 or PBS as a control, and, at the indicated time points, blood was withdrawn by cardiac puncture with an EDTA-coated syringe for analysis with flow cytometry and the animal was sacrificed.

Cell Sorting

Mouse $Ly6C^{low}$ or $Ly6C^{high}$ monocytes were sorted from Lin (CD3, CD19, NK1.1)$^-$ CD11b+, CD115+ gated cells using a Aria II FACS (BD) with 405, 488, 561 and 633nm laser lines. Human $CD14^{dim}$ monocytes were sorted from RBC lysed whole blood as described previously (Cros et al., 2010) into Opti-MEM (Life Technologies).

Analysis of Circulating Blood Ly6C^{low} Monocytes after Blocking CD11a

11 week old littermate male C57BL6 mice were warmed to 37°C and then either 4mg/kg IgG2a Rat isotype control (2A3; BD Pharmingen) or 4mg/kg rat anti-mouse CD11a (M17/4; BD Pharmingen) was injected intravenously via the tail vein in 100 μl 0.9% NaCl. 15 min later, 700-900μl blood was taken via cardiac puncture and the mouse was euthanized. RBC lysis was performed as above and the leukocytes were counted. Flow cytometry analysis was performed as above.

Immunofluorescence Staining and Confocal Microscopy of Fixed Samples

~15^4 Sorted human CD14^{dim} monocytes in HBSS were plated on 13mm glass coverslips that had been precoated overnight at 4°C with 3.5μg/ml human Fc-ICAM1 and 100 ng/ml recombinant human CSF1 (R&D systems) and blocked for 2 hr at room temperature with PBS-BSA 2% (w/v). Cells were incubated for 20 min at 37°C, 5% CO_2 and paraformaldehyde solutions were preheated to 37°C then the cells were fixed for 5 min with 3% paraformaldehyde in k-PIPES pH6.5 and 10 min 3% paraformaldehyde in Sodium Borate pH11 at room temperature, then washed in PBS. Autofluorescence was quenched by incubating in 0.1mg/ml $NaBH_4$ (sodium borohydride; Sigma) in PBS for 2 min. Cells were then permeabilized with PBS-BSA 1%- Triton X-100 0.1% for 5 min and washed with PBS-T. Following a 30 min incubation at room temperature with PBS-T, cells were incubated with anti-human CD11a (mAb 38) for 90 min and subsequently DyLight549 conjugated goat anti-mouse IgG2a and Phalloidin-AlexaFluor488 then mounted in Vectashield containing DAPI. Cells were imaged using a Leica SP5 with 405, 488 and 561 nm laser lines for DAPI, Phalloidin, and DyLight549 respectively, using sequential and spectral scanning to minimize cross channel bleed-through. A 63x 1.4 N.A. oil immersion objective was used to image the cells.

Multiplexed ELISA for In Vitro Cytokine Production

Lin- CD11b CD115$^+$ Ly6C$^+$ and Ly6C^{low} monocytes were sorted from the blood of mice by flow cytometry as described previously (Auffray et al., 2007) and above. Monocytes (0.1x10^6/ml) were incubated in medium (Opti-MEM) with or without the selective MEK inhibitor PD98059 (10 μM) for 45 min before addition of LPS (100 ng/ml; Sigma) or R848 (2 μg/ml; InvivoGen). Supernatants were collected after overnight incubation at 37°C. For cytokine measurements, plates were centrifuged, supernatant collected and stored at −80°C until analysis. IL1β, KC, CCL3, TNF and IL-6 cytokines were measured using the BioRad BioPlex murine cytokine kit according to manufacturer's instructions. Bead fluorescence emission was detected using the Luminex LX100 multiplex system (Luminex) and data analyzed using STarStation3.0 (Applied Cytometry) according to manufacturer's instructions.

SUPPLEMENTAL REFERENCES

Boscacci, R.T., Pfeiffer, F., Gollmer, K., Sevilla, A.I., Martin, A.M., Soriano, S.F., Natale, D., Henrickson, S., von Andrian, U.H., Fukui, Y., et al. (2010). Comprehensive analysis of lymph node stroma-expressed Ig superfamily members reveals redundant and nonredundant roles for ICAM-1, ICAM-2, and VCAM-1 in lymphocyte homing. Blood *116*, 915–925.

Ding, Z.M., Babensee, J.E., Simon, S.I., Lu, H., Perrard, J.L., Bullard, D.C., Dai, X.Y., Bromley, S.K., Dustin, M.L., Entman, M.L., et al. (1999). Relative contribution of LFA-1 and Mac-1 to neutrophil adhesion and migration. J. Immunol. *163*, 5029–5038.

Hemmi, H., Kaisho, T., Takeuchi, O., Sato, S., Sanjo, H., Hoshino, K., Horiuchi, T., Tomizawa, H., Takeda, K., and Akira, S. (2002). Small anti-viral compounds activate immune cells via the TLR7 MyD88-dependent signaling pathway. Nat. Immunol. *3*, 196–200.

Lee, S.L., Wesselschmidt, R.L., Linette, G.P., Kanagawa, O., Russell, J.H., and Milbrandt, J. (1995). Unimpaired thymic and peripheral T cell death in mice lacking the nuclear receptor NGFI-B (Nur77). Science *269*, 532–535.

Schaefer, B.C., Schaefer, M.L., Kappler, J.W., Marrack, P., and Kedl, R.M. (2001). Observation of antigen-dependent CD8+ T-cell/ dendritic cell interactions in vivo. Cell. Immunol. *214*, 110–122.

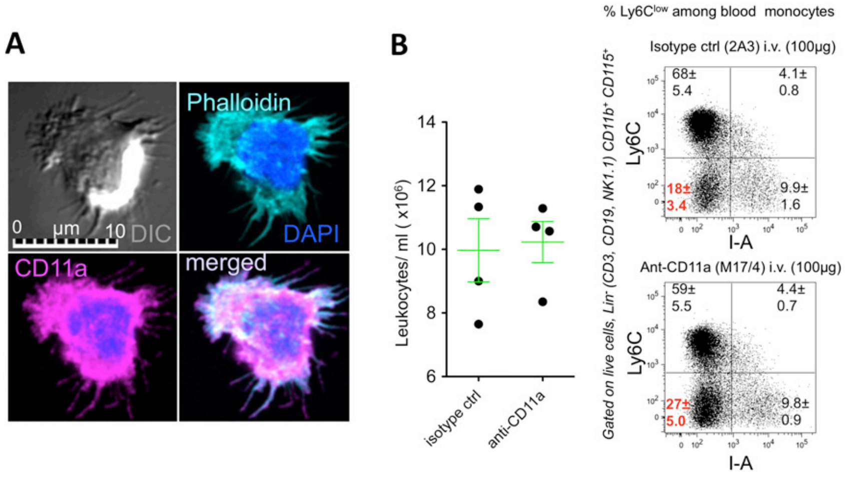

Figure S1. Phenotype of “Patrolling” Monocytes, Related to Figures 1 and 2

(A) In vitro staining of a human CD14^{dim} monocyte spread on an ICAM and CSF1 coated coverslip. (Gray – DIC, Cyan– Phalloidin, Magenta– α-CD11a, Blue – DAPI).

(B) Left - Leukocyte counts per ml for 11-wk old C57BL6 mice treated with isotype ctrl, or anti-CD11a Ab i.v. for 15 min. Right - representative dot plots (giving mean and SEM for each quadrant frequency) Ly6Clow, I-A- cell frequency is highlighted in red n = 4 mice per group.

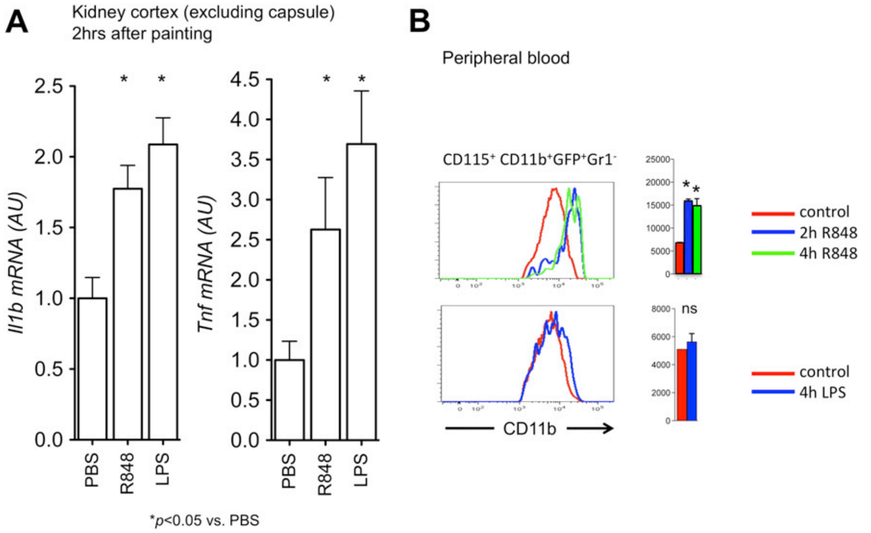

Figure S2. Response to R848 and LPS, Related to Figure 3

(A) Application of R848 or LPS to the kidney capsule induces inflammatory cytokines. qPCR for *Il1b* or *Tnf* mRNA in sub-capsular kidney cortex of *Cx3cr1*$^{-/+}$; *Rag2*$^{-/-}$; *Il2rg*$^{-/-}$ mice tissue treated in vivo by painting the kidney capsule with PBS, R848 (200 μg) or LPS (200 μg) for 5 hr n = 2-4 mice per condition. mRNA quantity was normalized to *Gapdh* and is expressed as fold change over PBS.

(B) CD11b expression on circulating Ly6C^{low} monocytes after kidney painting with either R848 (top) or LPS (bottom). Representative histograms are shown (left) and cumulative data mean ± SEM right, n = 3 for R848, n = 2 for LPS.

Cell

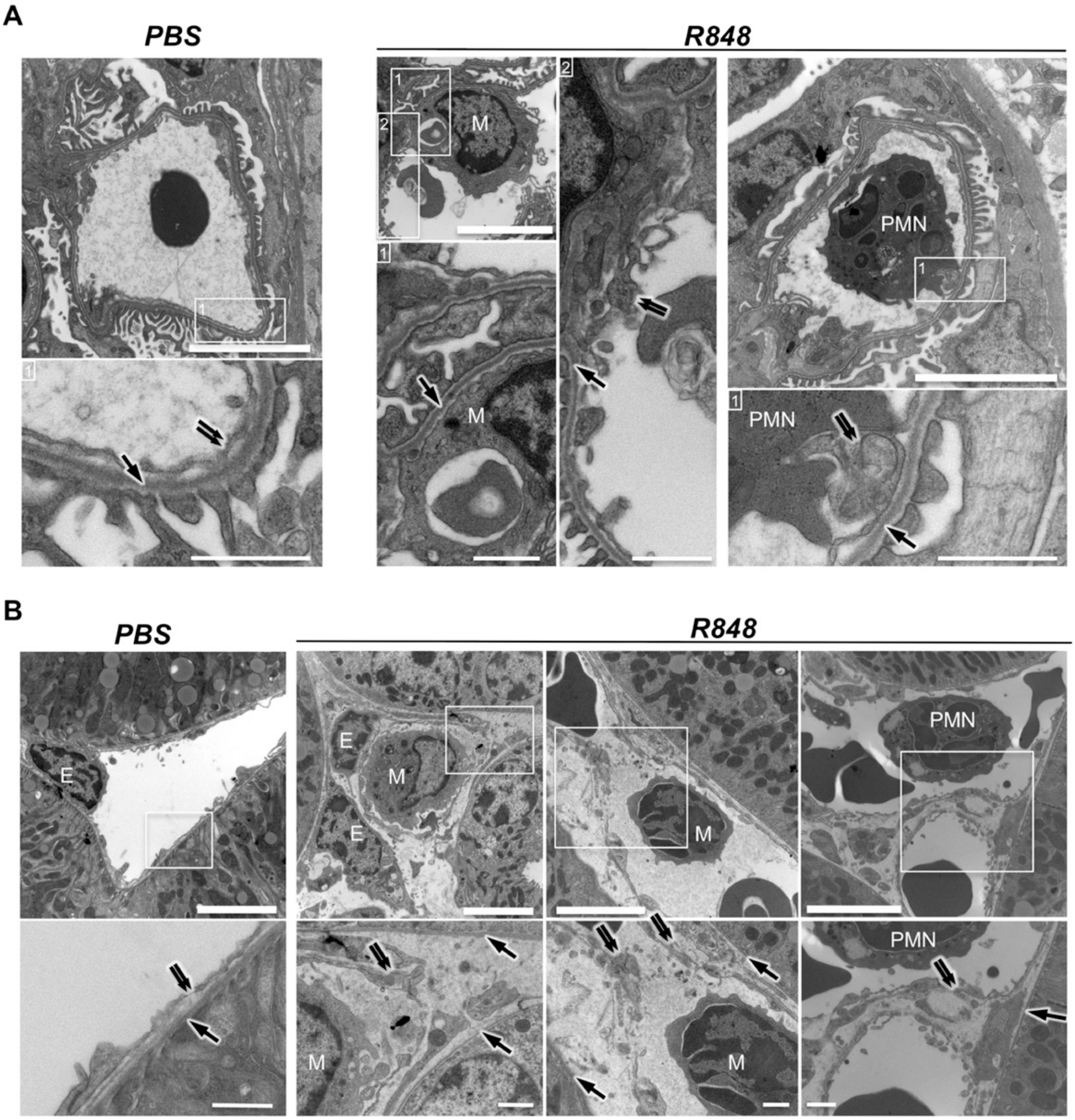

Figure S3. TEM of Kidney Capillaries 5 hr after Direct Application of PBS or R848 In Vivo, Related to Figures 4, 5, 6, and 7

Single arrow, basal lamina; double arrow, endothelium; M, shows mononuclear cell; PMN, polymorphonuclear cell; E, endothelial cell nucleus. White boxes indicate the high magnification areas.

(A) Glomerular capillaries from *Cx3cr1*$^{-/+}$*; Rag2*$^{-/-}$*; Il2rg*$^{-/-}$ mice. Left, PBS treated control glomerulus, endothelium is intact and close to basal lamina; Middle, R848 treated glomerular capillary containing mononuclear cell. Endothelium is missing and monocnuclear cell containing phagocytosed material sits directly against basal lamina (see higher magnifications); Right, PMN containing glomerular capillary with thickened area of endothelium.

(B) Peritubular capillaries from wild-type B6 mice. Left, PBS treated control peritubular capillary; 3rd from right, peritubular capillary containing mononuclear cell with necrotic and disrupted endothelium; 2nd from right, peritubular capillary containing mononuclear cell with necrotic and disrupted endothelium and expanded subendothelial space; 1st from right, peritubular capillary containing PMN with necrotic and disrupted endothelium and expanded subendothelial space. Images representative of 3 mice per condition. Thick scale bars = 5 μm, thin scale bars = 1 μm. Original magnification x15 000.
